# Serum concentrations of legacy, alternative, and precursor per- and polyfluoroalkyl substances: a descriptive analysis of adult female participants in the MIREC-ENDO study

**DOI:** 10.1186/s12940-024-01085-z

**Published:** 2024-06-10

**Authors:** M. M. Borghese, A. Ward, S. MacPherson, K. E. Manz, E. Atlas, M. Fisher, T. E. Arbuckle, J. M. Braun, M. F. Bouchard, J. Ashley-Martin

**Affiliations:** 1 Environmental Health Science and Research Bureau, Health Canada, Ottawa, ON Canada; 2 Department of Environmental Health Sciences, University of Michigan, Ann Arbor, MI USA; 3 Department of Epidemiology, Brown University, Providence, RI USA; 4 Institut national de la recherche scientifique, Laval, QC Canada

**Keywords:** Biomonitoring, Perfluorinated substances, Polyfluoroalkyl substances, Environmental chemicals

## Abstract

**Background:**

Several legacy and emerging per- and polyfluoroalkyl substances (PFAS) have been regulated around the world. There is growing concern over the proliferation of alternative PFAS, as well as PFAS precursors. Biomonitoring data for PFAS are critical for assessing exposure and human health risk.

**Methods:**

We collected serum samples from 289 adult female participants in a 2018–2021 follow-up study of the Maternal-Infant Research on Environmental Chemicals (MIREC) Canadian pregnancy cohort. Samples were analyzed for 40 PFAS using ultra-performance liquid chromatography–tandem mass spectrometry. For those compounds with > 50% detection, as well as the sum of these compounds, we describe serum concentrations and patterns of exposure according to sociodemographic and obstetrical history characteristics.

**Results:**

17 out of 40 PFAS were detected in > 50% of samples with 7 of these detected in > 97% of samples. Median [95th percentile] concentrations (µg/L) were highest for PFOS (1.62 [4.56]), PFOA (0.69 [1.52]), PFNA (0.38 [0.81]), and PFHxS (0.33 [0.92]). Geometric mean concentrations of PFOA and PFHxS were approximately 2-fold lower among those with more children (≥ 3 vs. 1), greater number of children breastfed (≥ 3 vs. ≤ 1), longer lifetime duration of breastfeeding (> 4 years vs. ≤ 9 months), and shorter time since last pregnancy (≤ 4 years vs. > 8 years). We observed similar patterns for PFOS, PFHpS, and the sum of 17 PFAS, though the differences between groups were smaller. Concentrations of PFOA were higher among “White” participants, while concentrations of N-MeFOSE, N-EtFOSE, 7:3 FTCA, and 4:2 FTS were slightly higher among participants reporting a race or ethnicity other than “White”. Concentrations of legacy, alternative, and precursor PFAS were generally similar across levels of age, education, household income, body mass index, and menopausal status.

**Conclusions:**

We report the first Canadian biomonitoring data for several alternative and precursor PFAS. Our findings suggest that exposure to PFAS, including several emerging alternatives, may be widespread. Our results are consistent with previous studies showing that pregnancy and breastfeeding are excretion pathways for PFAS.

**Supplementary Information:**

The online version contains supplementary material available at 10.1186/s12940-024-01085-z.

## Introduction

Per- and polyfluoroalkyl substances (PFAS) are a class of highly fluorinated chemicals with lipophobic and hydrophobic properties that are useful for manufacturing a wide range of consumer products, such as non-stick cookware, food packaging, personal care and beauty products, fire retardant foams, and carpet treatment applications [1–3]. These substances persist in the environment and several have been detected in human populations worldwide [4–7]. Among the general population, the primary sources of exposure are food, drinking water, and house dust [8–11]. Some of the compounds in this chemical class are biopersistent, with half-lives ranging from days to years [12, 13]; biomonitoring surveys have demonstrated that exposure to several of these compounds is ubiquitous among Canadians [5].

Epidemiological [2, 14–16] and experimental [15, 17–21] evidence broadly support deleterious associations between PFAS and health effects in humans. While there are thousands of PFAS [22], the majority of this evidence for human exposure is for two legacy PFAS, namely perfluorooctanoic acid (PFOA) and perfluorooctane sulfonate (PFOS). In the 2000s, a progressive phase out of PFOA and PFOS production began in the United States [2]. Since then, there has been considerable regulation on their production and use globally [23–25]. In Canada, risk management actions have been in place since 2010 and the manufacture, use, sale, offer for sale, or import of PFOA and PFOS, as well as products that contain them, have been prohibited since 2016 [26]. As a result, blood concentrations of PFOA, PFOS, along with perfluorohexane sulfonate (PFHxS), decreased on average in Canada by approximately 50–60% between 2007 and 2017 [27], with similar trends observed in the United States [28–31], Sweden [31], and Belgium [32]. This global phasing out of legacy PFAS has led to growing concern over the proliferation of alternative and precursor PFAS [14–16, 23, 24]; human biomonitoring data for some of these compounds is emerging [33], but is still limited. Several of these PFAS have been detected with increasing concentrations in Canadian freshwater environments since 2013 [34]. Timely Canadian biomonitoring data for these alternative and precursor PFAS are needed to better understand exposure and potential risks to human health and to evaluate the effectiveness of regulatory and risk management actions. It is necessary to characterize exposure not just generally, but across a range of socio-demographic and lifestyle factors in order to identify populations that may be disproportionately impacted by exposure to legacy, alternative and precursor PFAS.

In this paper, we use data from a 2018–2021 follow-up study of adult female participants from the Maternal-Infant Research on Environmental Chemicals (MIREC) pan-Canadian pregnancy cohort. Our objectives were to describe serum concentrations of 40 legacy, alternative, and precursor PFAS and to examine patterns of exposure according to sociodemographic and obstetrical history characteristics for those compounds with > 50% detection.

## Methods

### Study participants

In the MIREC study, 2001 pregnant participants in their first trimester were recruited between 2008 and 2011 from obstetric and prenatal clinics in 10 cities in Canada [35]. Participants were ineligible if they were < 18 years old, > 14 weeks gestation, reported illicit drug use or serious medical conditions, could not communicate in either English or French, or if they had known fetal abnormalities or a history of serious medical conditions (i.e., molar pregnancy, threatened spontaneous abortion, renal disease, epilepsy, collagen diseases, active and chronic liver disease, heart disease, serious pulmonary disease, cancer, or haematological disorders). In the MIREC-ENDO follow-up study in 2018–2021, a subset of participants from 8 of the initial 10 sites completed an in-person clinic visit that included physical measures (e.g., weight), questionnaires to collect sociodemographic and health history information, and biospecimen (e.g., blood and urine) collection. Both studies were approved by the Research Ethics Boards of Health Canada/Public Health Agency of Canada, Sainte-Justine University Hospital, as well as those of all MIREC-affiliated study sites. Informed consent was obtained from all participants. Of the participants recruited for the MIREC study (*n* = 2001), this analysis was restricted to the subset of participants that attended a clinic visit for the follow-up study and provided a blood sample [36]. Overall, 308 participants attended a clinic visit and 289 of these participants provided a blood sample.

### Blood collection, laboratory analysis, and quality control

Blood samples were collected using 10-ml sterile vacutainer tubes. Within 2 h of the blood draw, samples were centrifuged to separate serum and aliquoted into smaller cryovials and stored at − 80 °C. Analysis of 40 legacy, alternative and precursor PFAS (Supplemental Table 1) was completed in 2022 by AXYS Analytical Services Ltd. (Sidney, BC, Canada) according to AXYS method MLA-110 (revision 2, version 12). The laboratory is ISO 17025 accredited by the Canadian Association for Laboratory Accreditation. This analysis included 11 perfluorinated carboxylic acids, 8 perfluorinated sulfonic acids, 6 fluorotelomers (3 carboxylic acids and 3 suflonic acids), 7 perfluorooctane sulfonamide-based precursors, 2 prominent PFOA replacement compounds (HFPO-DA [GenX] and ADONA) as well as 3 other per- and polyfluoroether carboxylates (NFDHA, PFMPA, PFMBA), 2 chlorinated polyfluorinated ether sulfonates (9Cl-PF3ONS [i.e., 6:2 Cl-PFESA], and 11Cl-PF3OudS [i.e., 8:2 Cl-PFESA]), and 1 polyfluoroether sulfonate. After spiking with isotopically labeled recovery standards (^13^C_3_-PFBA, ^13^C_2_-PFHxA, ^13^C_4_-PFOA, ^13^C_5_-PFNA, ^13^C_2_-PFDA, ^18^O_2_-PFHxS, ^13^C_4_-PFOS, ^13^C_2_-D_4_-6:2-FTS), samples were extracted and cleaned up by solid phase extraction. The extracts were then analyzed by ultra-performance liquid chromatography on a reversed phase C18 column using a solvent gradient. The column was coupled to a triple quadrupole mass spectrometer operated in the multiple reaction monitoring mode, using negative electrospray ionization. Final sample concentrations were determined by isotope dilution/internal standard quantification and adjusted for percent recovery. After initial calibration, a mid-level calibration was analyzed after every 12 h. Limits of detection (LOD) were assigned for each compound by determining the concentration at which the peak area was equivalent to 3.0 times the estimated chromatographic noise height. The median and inter-quartile range of LODs for blank samples for all compounds across 15 batches are presented in Supplemental Table 2.

Two types of QC samples were used to assess PFAS recovery: water and serum matrix spikes. A blank water sample spiked with isotope labeled PFAS was analyzed in each of the 15 analytical batches (approximately 20 samples/batch). The percent recovery was within the acceptable range of 70–130% for most surrogates (Supplemental Table 3), with the exception of D_7_-N-MeFOSE (used to quantify N-MeFOSE) for which 12/15 batches exceeded 130% (median = 149%). Percent recovery of the native compound N-MeFOSE was lower, but still within the acceptable range. The second type of QC sample analyzed by the laboratory was spiked reference serum. In each batch, a low and high concentration of unlabeled and labeled PFAS were spiked into the serum. Samples were deemed acceptable if the percent recovery ranged from 70 to 130% (Supplemental Table 4); percent recoveries were within this range for most PFAS. However, for NFDHA the percent recovery was often < 70% (10/15 batches for low spike and 11/15 batches for high spike) and the coefficient of variation of percent recoveries was high (48% and 33.5% for low and high spike samples, respectively). For 3:3 FTCA the percent recovery was < 70% for all batches. Therefore, the estimated concentrations of NFDHA and 3:3 FTCA should be interpreted cautiously.

We examined PFAS concentration data for potential outliers and batch effects. We determined that concentrations of 6:2 FTS were extremely high in serum samples from one batch (range 11–269 µg/L). The percent recoveries for 6:2 FTS in the spiked reference serum for this batch were high, but within the acceptable range (118% and 129% for the high and low spikes, respectively), while the percent recovery of the isotopically labelled internal standard in the water QC sample was low (59.9%). Results for the other 39 PFAS in this batch were similar to the other batches. We speculate that this is the result of an unknown contamination during processing of serum samples for this batch. Data for 6:2 FTS from this batch were removed from this analysis, which is reflected in the difference in sample sizes for 6:2 FTS (*n* = 269) vs. other PFAS (*n* = 289).

### Covariate data collection

Participants completed detailed questionnaires in the first trimester and at follow-up to provide sociodemographic information. At the first trimester, participants reported their country of birth (Canada vs. other) as well as race and ethnicity. Participants self-reported their race and ethnicity from the following options: White, Chinese, South Asian, Black, Filipino, Southeast Asian, Latin American, Arab, West Asian, Japanese, Korean, Aboriginal. In this analysis, participants who exclusively identified their race or ethnicity as “White” were coded as being White, while participants selecting any other category or combination of categories were coded as “Other”. At the follow-up visit, participants provided their age, household income (≤ 60,000, 60,001–100,000, or ≥ 100,000 in $CAD), level of education (2-year college diploma or less vs. bachelor’s degree or higher), number of children (1, 2, ≥ 3), cigarette smoking status (never, former, current), menopausal status (premenopausal, peri/post-menopausal, or unknown because of hormonal contraceptive use), number of children breastfed (≤ 1, 2, or ≥ 3) and the lifetime duration of breastfeeding (sum of duration of breastfeeding for each child as ≤ 2, > 2–4, or > 4 years). Time since last pregnancy was determined from participants’ self-reported obstetrical history (≤ 4, > 4–8, > 8 years). Body mass index (kg/m^2^) at follow-up was derived from measured height and weight and was categorized according to World Health Organization classifications [37] (under/normal weight, overweight, and obese with cut-offs at 25 and 30 kg/m^2^, respectively).

### Statistical analysis

Statistical analysis was performed in R v. 3.6.2 (R Core Team, 2020. R Foundation for Statistical Computing, Vienna, Austria) and SAS Enterprise Guide 7.1 (SAS institute, Cary, NC.). Concentrations were blank-corrected by subtraction and negative values were replaced with one-half of the smallest positive observed value. Non-detect values were replaced with the batch-specific blank LOD/√2 [38] to facilitate comparisons with previous analyses using this same method. While this is the most common method used to handle non-detect values, it has been criticized as potentially introducing bias. Therefore, we also generated summary statistics using a maximum likelihood estimation approach with a lognormal distribution and compared findings using these two methods. We report geometric means and 95% confidence intervals (95% CI) for all compounds and calculated Spearman correlation coefficients for PFAS with a detection rate greater than 50%; we report Spearman correlations with 6:2 FTS separately using the lower sample size as a result of excluding one batch of data. We used general linear models with log-transformed PFAS concentrations as the dependent variable, as well as the arithmetic sum concentration of 17 PFAS (Σ17PFAS) with > 50% detection, to calculate the geometric mean (95% CI) concentrations within strata of sociodemographic and obstetrical history characteristics and used ANOVA to test for differences between group-specific means. When the overall group effect was statistically significant (p-value < 0.05), we conducted pairwise comparisons using the Bonferroni method. We evaluated model diagnostics by checking normality and homoscedasticity of the residuals. PFAS data were log-transformed to satisfy model assumptions. The assumption of normality of residuals was satisfied for nearly all PFAS models, with the exception of N-MeFOSE; therefore, we used the non-parametric Kruskal Wallis method for N-MeFOSE only.

## Results

Similar to the original MIREC cohort, MIREC-ENDO participants tended to be White, born in Canada, and of moderate to high socioeconomic status (Table 1). 17 out of 40 PFAS were detected in > 50% of samples (Table 2); 7 of these were detected in > 97% of samples. Geometric mean concentrations of these 17 PFAS ranged from 0.001 (PFMBA) to 1.669 (PFOS) µg/L and was 4.089 µg/L for the Σ17PFAS. Geometric means were similar in analyses using the maximum likelihood estimation method (Supplemental Table 5). Among those compounds with < 50% detection, 95th percentile concentrations ranged from 0.002 (PFEESA and PFDoS) to 0.174 (PFTeDA) µg/L. Spearman correlation coefficients were generally highest among PFOA, PFHxS, PFOS, PFNA, PFDA, and PFUDA, with correlations as high as 0.65 (Supplemental Fig. 1). Correlations between other compounds ranged from − 0.24 to 0.30.

**Table 1:** Sociodemographic characteristics of adult female participants in the MIREC-ENDO study (2018–2021; *n* = 289)

	*n*	%
Age		
32–39	82	28.4
40–44	111	38.4
45–49	71	24.6
50 and over	25	8.7
Race and ethnicity		
White	258	89.3
Other	26	9.0
Missing	5	1.7
Country of birth		
Canada	248	85.8
Other	36	12.5
Missing	5	1.7
Body mass index (kg/m^2^)		
< 25	122	42.2
25–29	78	27.0
≥ 30	57	19.7
Missing	32	11.1
Smoking status		
Never/not at all	192	66.4
Former smoker	75	26.0
Current smoker	22	7.6
Menopausal status		
re-menopausal	172	59.5
Peri- and post-menopausal	54	18.7
Using contraceptives	46	15.9
Missing	17	5.9
Parity		
1	32	11.1
2	148	51.2
≥ 3	93	32.2
Missing	16	5.5
Time since last pregnancy (years)		
≤ 4	33	11.4
> 4–8	98	33.9
> 8	142	49.1
Missing	16	5.5
Number of children breastfed		
≤ 1	41	14.2
2	123	42.6
≥ 3	86	29.8
Missing	39	13.5
Lifetime duration of breastfeeding (years)		
≤ 2	121	41.9
> 2–4	90	31.1
> 4	39	13.5
Missing	39	13.5
Level of education		
College diploma or less	70	24.2
Some university or more	219	75.8
Annual household income ($CAD)		
≤ 60 000	23	8.0
60 001 to 100 000	56	19.4
More than 100 000	204	70.6
Missing	6	2.1

**Table 2:** Descriptive statistics for per- and polyfluoroalkyl substances (µg/L) and the sum of 17 PFAS with > 50% detection in adult females from the MIREC-ENDO study (2018–2021)

	*n*	% > LOD^1^	25th percentile	Median	75th percentile	95th percentile	Geometric mean (95% CI)
Σ17PFAS	289	-	2.914	4.113	5.118	9.609	4.089 (3.835, 4.361)
PFOS	289	100	1.128	1.617	2.311	4.563	1.669 (1.558, 1.788)
PFHxS	289	100	0.195	0.332	0.521	0.920	0.315 (0.290, 0.343)
N-EtFOSE	289	99.7	0.001	0.001	0.008	0.050	0.003 (0.002, 0.003)
PFNA	289	99.3	0.274	0.376	0.518	0.814	0.363 (0.338, 0.389)
PFOSA	289	99.0	0.001	0.001	0.002	0.010	0.002 (0.002, 0.002)
PFOA	289	97.6	0.442	0.692	0.946	1.517	0.504 (0.437, 0.581)
7:3 FTCA	289	97.2	0.004	0.004	0.155	1.499	0.024 (0.019, 0.031)
6:2 FTS	269	85.1	0.002	0.011	0.061	0.264	0.015 (0.012, 0.019)
PFPeA	289	79.6	0.001	0.019	0.038	0.077	0.009 (0.007, 0.011)
PFDA	289	75.4	0.080	0.146	0.221	0.421	0.140 (0.130, 0.151)
PFBA	289	71.6	-	0.093	0.212	0.333	0.090 (0.078, 0.104)
N-MeFOSE	289	67.8	-	0.009	0.015	0.042	0.006 (0.005, 0.007)
PFHpS	289	66.1	-	0.018	0.041	0.076	0.012 (0.010, 0.014)
PFUDA	289	60.6	-	0.100	0.179	0.310	0.086 (0.077, 0.098)
PFMBA	289	56.4	-	0.001	0.002	0.004	0.001 (0.001, 0.001)
4:2 FTS	289	54.0	-	0.004	0.008	0.025	0.004 (0.003, 0.004)
N-MeFOSAA	289	51.2	-	0.008	0.036	0.119	0.013 (0.011, 0.015)
8:2 FTS	289	49.5	-	-	0.017	0.128	-
HFPO-DA	289	47.1	-	-	0.007	0.028	-
PFHpA	289	39.1	-	-	0.062	0.125	-
N-EtFOSA	289	38.8	-	-	0.006	0.012	-
N-MeFOSA	289	38.4	-	-	0.008	0.016	-
PFHxA	289	35.6	-	-	0.057	0.130	-
PFBS	289	32.5	-	-	0.006	0.034	-
PFPeS	289	22.1	-	-	-	0.031	-
PFMPA	289	20.4	-	-	-	0.016	-
ADONA	289	15.9	-	-	-	0.008	-
9Cl-PF3ONS	289	15.9	-	-	-	0.029	-
N-EtFOSAA	289	13.1	-	-	-	0.019	-
PFDS	289	11.1	-	-	-	0.006	-
PFTeDA	289	10.4	-	-	-	0.174	-
PFDoA	289	8.7	-	-	-	0.067	-
PFNS	289	7.6	-	-	-	0.005	-
5:3 FTCA	289	6.6	-	-	-	0.141	-
PFTrDA	289	6.2	-	-	-	0.117	-
PFEESA	289	5.5	-	-	-	0.002	-
3:3 FTCA^2^	289	2.8	-	-	-	-	-
11Cl-PF3OUdS	289	2.1	-	-	-	-	-
NFDHA^2^	289	1.4	-	-	-	-	-
PFDoS	289	0.7	-	-	-	-	-

LOD – limit of detection; Σ17PFAS – concentration sum of 17 PFAS with > 50% detection

Geometric means were not calculated for chemicals with > 50% of values below the limit of detection

^1^LODs are provided in Supplemental Table 2

^2^Interpret with caution due to low percent recovery

**Fig. 1:**
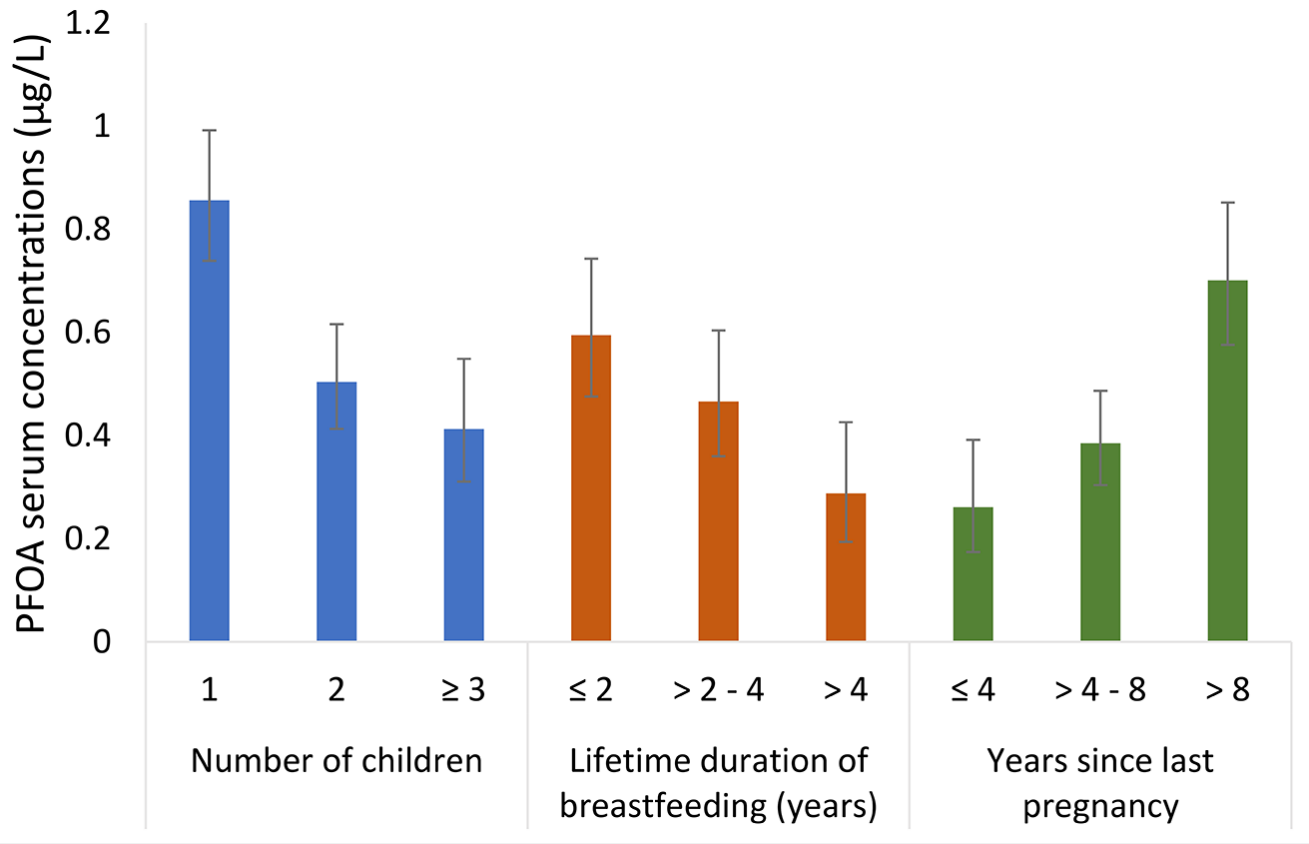
Geometric mean (95% CI) serum concentrations of PFOA stratified by number of children, lifetime duration of breastfeeding, and years since last pregnancy in the MIREC-ENDO study (2018–2021)

**Fig. 2:**
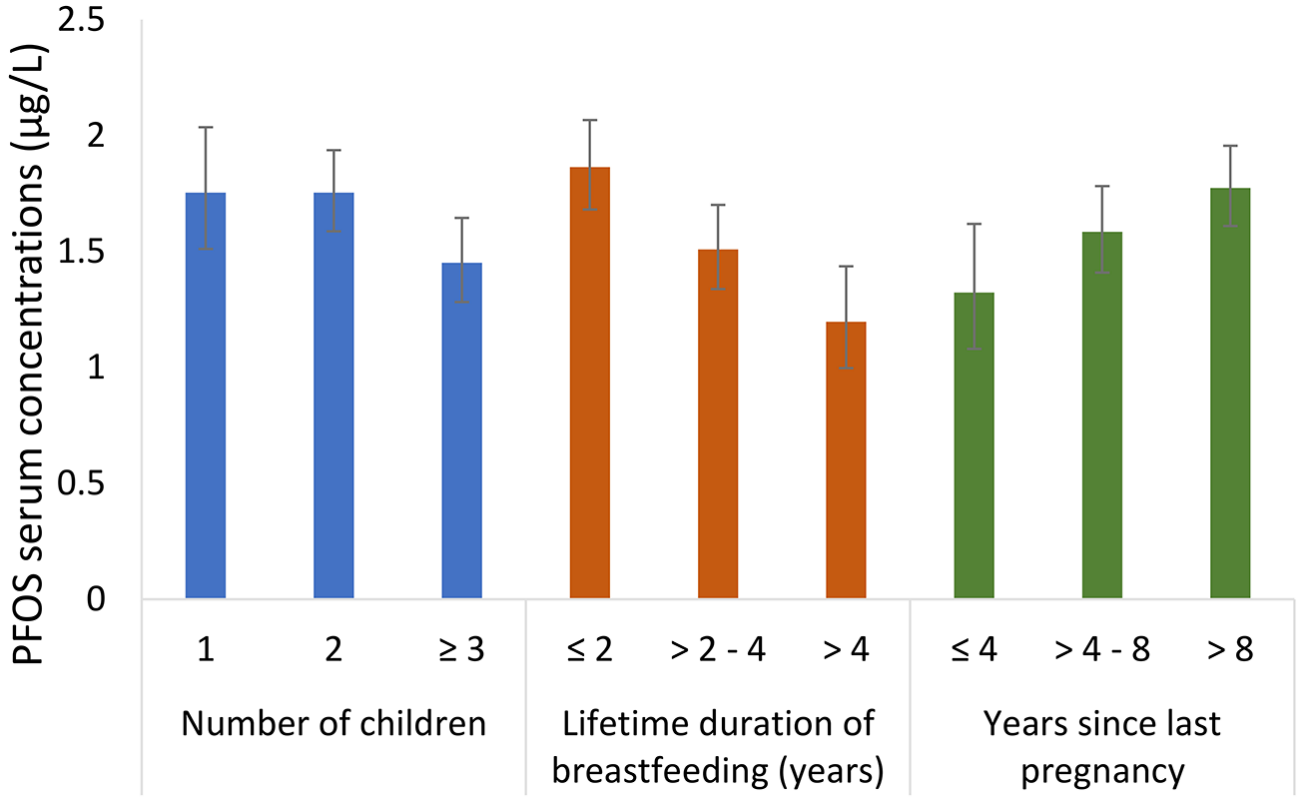
Geometric mean (95% CI) serum concentrations of PFOS stratified by number of children, lifetime duration of breastfeeding, and years since last pregnancy in the MIREC-ENDO study (2018–2021)

**Fig. 3:**
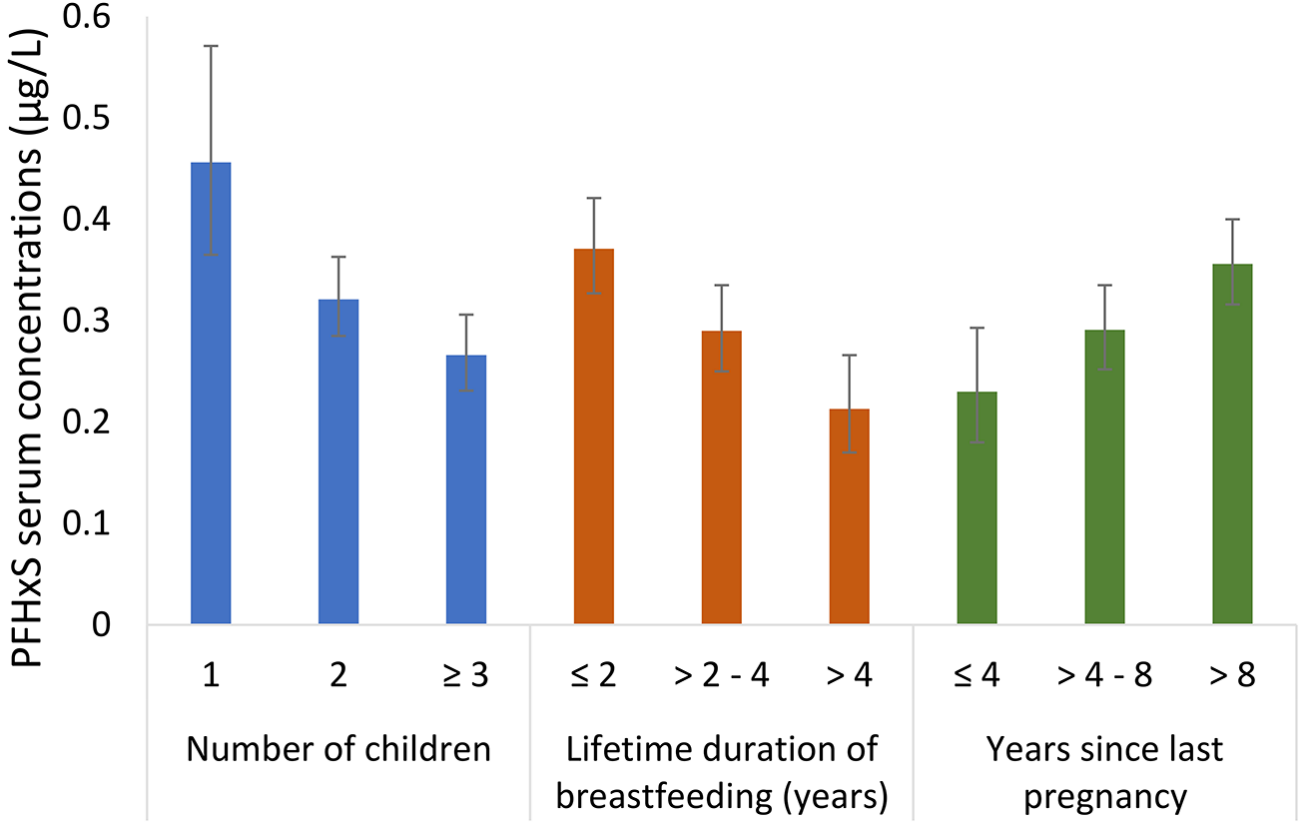
Geometric mean (95% CI) serum concentrations of PFHxS stratified by number of children, lifetime duration of breastfeeding, and years since last pregnancy in the MIREC-ENDO study (2018–2021)

**Fig. 4:**
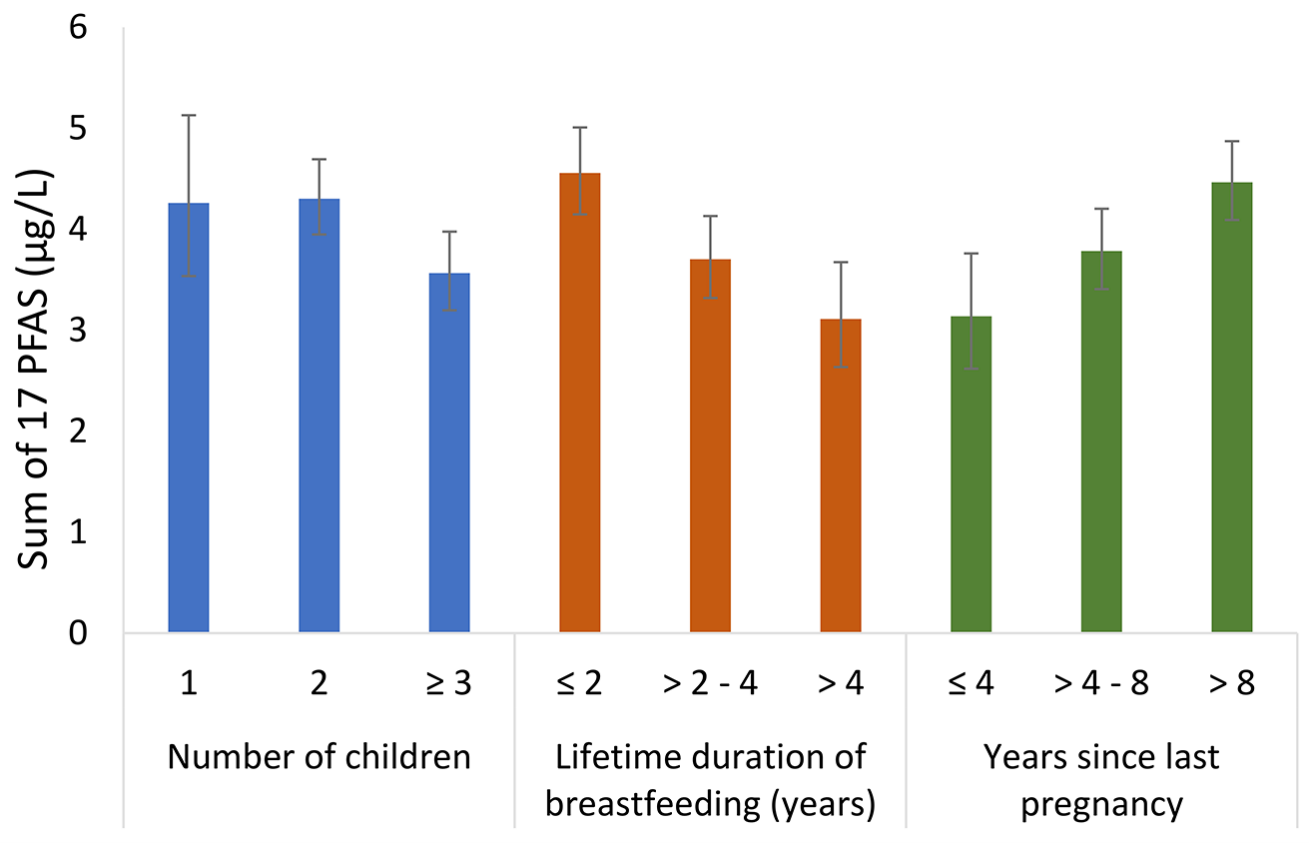
Geometric mean (95% CI) of sum of 17 PFAS with > 50% detection stratified by number of children, lifetime duration of breastfeeding, and years since last pregnancy in the MIREC-ENDO study (2018–2021)

Geometric mean concentrations of the 17 PFAS with > 50% detection, and their sum, across categories of sociodemographic and obstetrical history characteristics are presented in Supplemental Tables 6–14. Concentrations of PFOA, PFOS, PFHxS, and Σ17PFAS were consistently lower among participants with more children, greater number of children breastfed, shorter time since last pregnancy (Figs. 1, 2, 3 and 4 and Supplemental Tables 6, 7, and 14), as well as longer lifetime duration of breastfeeding. We observed approximately 2-fold differences in geometric mean concentrations of PFOA and PFHxS when comparing the highest vs. lowest categories of these factors. We observed a similar pattern for PFHpS, but the absolute differences were 0.013 µg/L or lower. For PFOS and Σ17PFAS, the differences in serum concentrations were small across categories of parity.

Overall, PFAS concentrations were generally similar across levels of age, education, household income, body mass index, and menopausal status; age, BMI, and income were inversely associated with some PFAS concentrations though infrequently and with non-monotonic patterns. Participants who self-identified their race and ethnicity as being exclusively White vs. Other had higher concentrations of PFOA (geometric mean = 0.538 vs. 0.322 µg/L); they also had lower concentrations of N-MeFOSE, N-EtFOSE, 7:3 FTCA, and 4:2 FTS but these differences in geometric mean ranged from 0.002 to 0.032 µg/L. Concentrations of PFBA were 2-fold higher among never/former vs. current smokers; however, there were few current smokers in this sample and the geometric mean for this group was imprecise. Participants born outside of Canada had slightly higher concentrations of PFDA (geometric mean = 0.177 vs. 0.136 µg/L) than those born in Canada.

## Discussion

We provide the first Canadian biomonitoring results for several alternative, and precursor PFAS using data from a 2018–2021 follow-up study of a pan-Canadian pregnancy cohort. Including legacy compounds, 17 out of 40 PFAS were detected in > 50% of samples, with 7 detected in > 97% of samples. To our knowledge, 10 of these compounds have not previously been measured in Canadians (N-EtFOSE, PFOSA, 7:3 FTCA, 6:2 FTS, PFPeA, N-MeFOSE, PFHpS, PFMBA, 4:2 FTS, N-MeFOSAA).

Our finding that serum concentrations of several legacy PFAS were markedly lower with greater number of children birthed and longer history of breastfeeding is in line with previous research that has identified pregnancy and lactation as prominent excretion pathways for PFAS [39–43]. This is also consistent with the moderate-to-strong correlations that we observed for these PFAS. The differences in PFOS concentrations were small across categories of parity but this is consistent with results from a meta-analysis of 39 studies demonstrating that PFOS is less efficiently transferred across the placenta than both PFOA and PFHxS [44]. PFOS has a stronger binding affinity for albumin [45] which may result in less free PFOS available for transplacental transfer. Six PFAS (PFOA, PFOS, PFHxS, PFNA, PFBS and PFHxA) were detected in human milk samples from a previous analysis of participants in this cohort [46] which supports the biological plausibility of the differences that we observed with breastfeeding history. We found a similar pattern of differences for PFNA across obstetrical history characteristics, but did not examine potential differences in concentrations for PFBS or PFHxA because the detection frequencies were < 50%. The current study has one of the longest follow-up times with complete obstetrical history data which allowed us to examine differences across a longer time since last pregnancy. In their analysis of interpregnancy intervals in the Danish National Birth Cohort, Bach et al. (2018) showed that concentrations of several legacy PFAS increased at 2–4 years post-pregnancy, and either remained stable or declined between 4 and 6 years post-pregnancy [47]. Our results suggest that legacy PFAS continue to bioaccumulate beyond 4–6 years post-pregnancy, with the largest differences observed > 8 years later. Future studies should consider obstetrical history characteristics as confounders of potential associations between PFAS and health outcomes influenced by parity and breastfeeding.

**Fig. 5:**
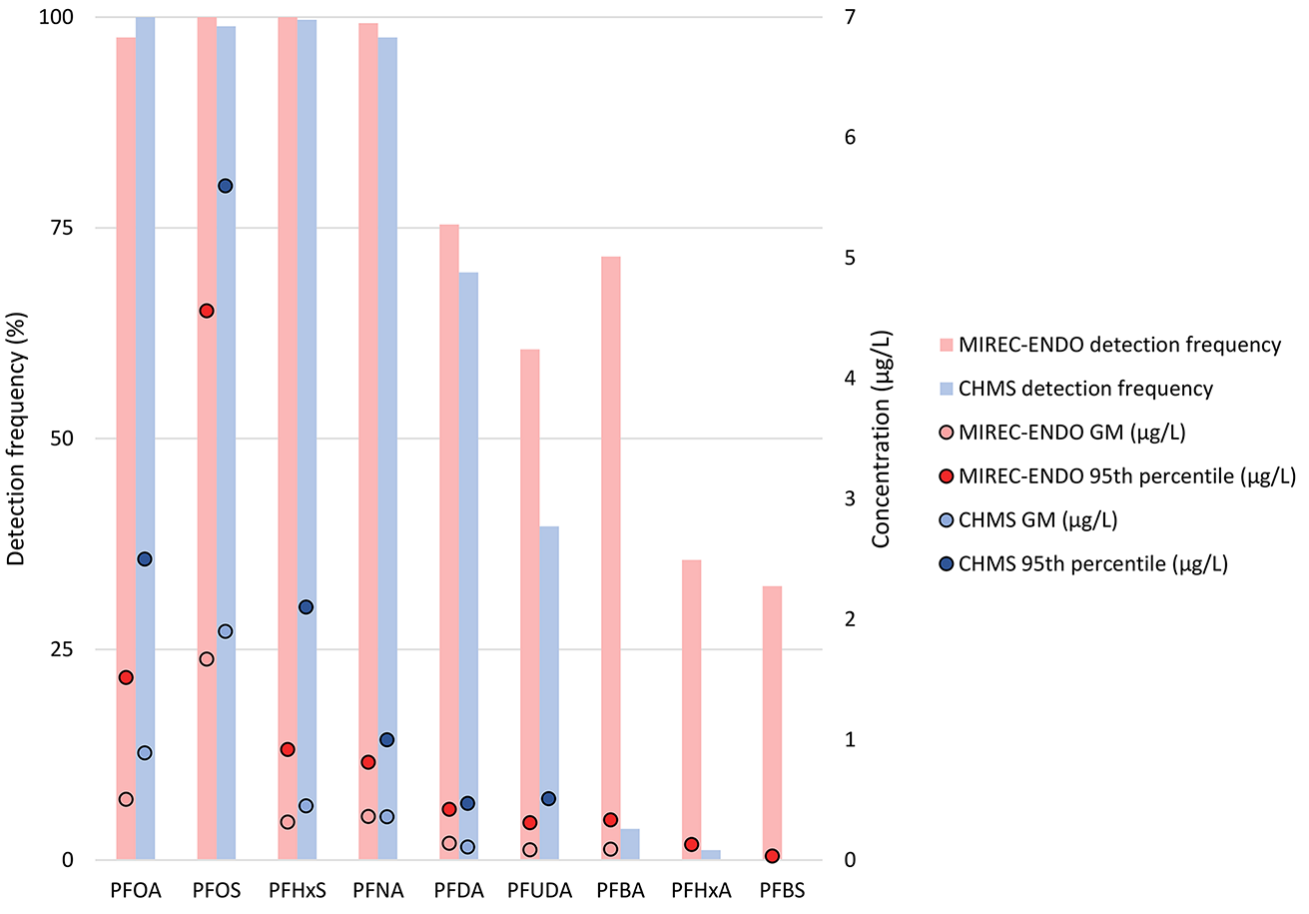
Detection frequencies, geometric means (GM) and 95th percentile concentrations of PFAS measured in MIREC-ENDO (2018–2021) and females aged 32–56 from the 2018–2019 Canadian Health Measures Survey (CHMS)

There are both similarities and differences between our results and data on 9 PFAS among females aged 32–56 from the 2018–2019 Canadian Health Measures Survey (CHMS) [3] (Fig. 5). Detection frequencies and concentrations for PFNA and PFDA in our study are in line with those from the CHMS. While detection frequencies were similarly high, both geometric mean and 95th percentile concentrations of PFOA, PFOS, and PFHxS are lower in the current study than those measured in the CHMS. The higher detection rates for PFUDA, PFBA, PFHxA in our study compared to the CHMS is likely because of the lower LODs in our study. For PFBA, the detection rate is considerably higher in our study (71.6%) than in the CHMS (3.7%), despite a higher LOD in our study. The geometric mean concentration of PFBA in our study is higher than the LOD in the CHMS, and the 75th percentile (0.212 µg/L) is considerably higher than LODs in both our study and the CHMS. It is not clear why we observed higher concentrations of PFBA for a portion of participants in our study.

While not included in previous cycles of the CHMS, some of the PFAS reported here have been measured in the US National Health and Nutrition Examination Survey (NHANES) [48]; we compare our findings to those among adult females using the most recent available cycle of NHANES for each PFAS. Detection frequencies and concentrations for PFHpA in our study were similar to those in NHANES (2013–2014). Although well detected our study and NHANES (2017–2018), concentrations of PFHpS were approximately 10-times lower in the current study, which is lower than the LOD in NHANES. PFDoA and PFHxA may have been more frequently detected in our study because of the lower LODs used in our study vs. NHANES (0.053 to 0.076 vs. 0.1 µg/L); for PFDoA, the 95th percentile in our study was below the LOD used in NHANES (2015–2016) while for PFHxA it was slightly higher than the LOD used in NHANES (2013–2014). While analytical differences likely contribute to these discrepancies in detection frequencies and concentrations, blood concentrations of several PFAS have historically differed between nationally representative surveys of Americans and Canadians [48, 49].

While not measured in blood in either the CHMS or NHANES, PFDS and PFTeDA were analyzed in human milk samples in the MIREC cohort collected between 2008 and 2011 but were not detected in any samples [50]; these two compounds were detected in 10–11% of samples in the current analysis. Canadian human biomonitoring data for PFPeA are not available for comparison, but the detection frequency in our study is higher than in two previous American and German studies. PFPeA was detected in 25% of participants from a small sample of residents from Indiana, USA in 2020 using a lower LOD (0.001 µg/L) than in the current study [51]. PFPeA was also measured in a study of the German general population (2009–2017) where it was detected in 4% of samples, though with a LOD 10-times higher than the current study (0.5 µg/L) [52]. The 95th percentile (0.077 µg/L) observed for PFPeA in our study was below the LOD used in the German study.

Compounds such as HFPO-DA (i.e., Gen-X), ADONA, 9Cl-PF3ONS (i.e., 6:2 Cl-PFESA or F53-B major), and 11Cl-PF3OUdS (i.e., 8:2 Cl-PFESA or F-53B minor) are increasingly being used as alternatives or replacements for PFOA and PFOS [53–56]. Human biomonitoring studies of the German [57] and American [48] general populations, pregnant people in China [41], as well as residents living near the Cape Fear river basin in North Carolina [58] (where HFPO-DA was found in high concentrations in drinking water [56]) have not detected HFPO-DA in serum. Similarly, the majority of human biomonitoring studies conducted worldwide in populations without an occupational source of exposure have rarely detected ADONA [41, 48, 57, 59]. In a German study of residents living near a fluorochemical production plant, ADONA was detected in up to 28% of participants living near the site or with drinking water sourced downstream in 2009, but in only 2% of participants in 2015 [60]. ADONA was not detected among participants living in an unexposed community 80 km away from the site at either time point [60]. The detection limits for HFPO-DA and ADONA used in these studies are 30 to 260 times (0.1 to 0.8 µg/L) and 10 to 250 times (0.01 to 0.25 µg/L) higher, respectively, than the LODs in our study. While HFPO-DA and ADONA were detected in 47% and 16% of participants in our study, respectively, the concentrations that we observed are much lower than the reporting limits used in previous work [41, 48, 57–60].

9Cl-PF3ONS and 11Cl-PF3OUdS are the primary components of F-53B [61], a industrial product used primarily in China as an alternative for PFOS [55] as a mist suppressant in the chrome plating industry [54]. Non-occupational exposure to 9Cl-PF3ONS, and to some extent 11Cl-PF3OUdS, appears to be widespread in the general population in China as measured in both serum [41, 55, 62–65] and human milk [66–69]. There is growing concern that these compounds could become globally dispersed environmental contaminants [70, 71]. They have been detected in serum of polar bears from Hudson Bay and the Beaufort Sea [72] as well as livers of marine mammals from Greenland [73, 74] and Sweden [74]. Human biomonitoring studies outside of China have recently begun to detect 9Cl-PF3ONS at low concentrations, including among Swedish adolescents in 2016–2017 (5% detection with LOD = 0.005–0.067) [70], and now in Canada as per the current study (15% detection with median LOD of 0.005). These infrequent and low-level detections outside of China may be explained by the longer half-life of Cl-PFESAs (up to 15.3 years [65]) compared to the shorter suspected half-lives for HFPO-DA [58, 75, 76] and ADONA [77]. Studies using less sensitive methods (LODs ranging from 0.1 to 0.25 µg/L) have not detected 9Cl-PF3ONS in the American general population in 2017–2018 [78] as well as 9Cl-PF3ONS or 11Cl-PF3OUdS in the German general population in 2009–2019 [57]. 11Cl-PF3OUdS was detected in 2% of participants in the current study. Future biomonitoring studies should consider measuring 9Cl-PF3ONS and 11Cl-PF3OUdS using sensitive methods to continue to monitor potential trends in exposure.

In Europe, 6:2 FTS is a common alternative to PFOS in the metal plating industry [79] and has been found in surface water globally [80], including in Canada [34]. In the environment, 6:2 FTS transforms into PFHxA [79]. Concentrations of both 6:2 FTS and PFHxA were low in the majority of participants in our study, but the 95th percentiles of both compounds were markedly higher relative to the median (0.264 and 0.130 µg/L). This may indicate that some individuals have unique sources of exposure to 6:2 FTS. Whole blood, rather than serum, may be the ideal matrix for measuring PFHxA [81] which could explain the lower detection frequency for PFHxA in the current study. Two other fluorotelomers – 7:3 FTCA and 4:2 FTS – measured in this study were detected in > 50% of samples. 8:2 FTS was detected in nearly 50% of samples, and 5:3 FTCA was infrequently detected. Concentrations of 4:2 FTS were low across the distribution, while in contrast the 95th percentiles of both 8:2 FTS and 5:3 FTCA were higher relative to the median (0.128 and 0.141 µg/L), while the 95th percentile for 7:3 FTCA was close to that for PFOA (1.499 µg/L). 4:2, 6:2, and 8:2 FTS were not detected in a 2009–2019 German general population study [57], though the detection limits were higher than the concentrations observed in the current study (0.25–0.50 µg/L). The high variability in LODs among different biomonitoring studies precludes comparison of detection rates for compounds that are rarely measured.

Several of the PFAS analyzed in the current study are sulfonamide-based precursors for the legacy PFAS. N-MeFOSAA and N-MeFOSA are oxidation products of N-MeFOSE (used in textile and carpet products) while N-EtFOSAA and N-EtFOSA are oxidation products of N-EtFOSE (used in paper and packaging products) [1, 82]. Both N-MeFOSE and N-EtFOSE transform into PFOSA which transforms into PFOA and PFOS as the terminal moieties [1, 83, 84]. Generally, human biomonitoring studies from the United States [48], Germany [57], and Sweden [70] have found low levels of detection and concentrations of sulfonamide-based precursors. Median concentrations of N-MeFOSAA, N-EtFOSAA, and PFOSA declined among the general American population from 0.30, 0.70, and 0.70 µg/L, respectively, in 1999–2000 to 0.10 µg/L for all three compounds in 2011–2012 [48]; N-MeFOSAA was also analyzed in subsequent cycles of NHANES until 2017–2018, and concentrations appear to be stable since then. In another study of the general German population between 2009 and 2019, N-MeFOSAA was detected in 2 out of 100 samples, while N-EtFOSAA, N-MeFOSA, N-EtFOSA, and PFOSA were not detected [57]; however, the detection limits (0.25–0.50 µg/L) were higher than the 90th percentile in NHANES [48]. A 2016–2017 nationally representative Swedish study detected N-MeFOSAA in 9% of blood samples provided by adolescents using LODs similar to the current study [70]. In our study, N-MeFOSAA was detected in > 50% of samples with low concentrations across most of the distribution; the 95th percentile in our study (0.119 µg/L) from 2018 to 2021 was similar to the median in NHANES in 2017–2018. N-MeFOSE was detected at slightly higher frequency, but at similarly low concentrations. N-EtFOSE and PFOSA were detected in > 99% of samples, while N-EtFOSAA, N-EtFOSA, and N-MeFOSA were infrequently detected, though all at low concentrations. Our results are generally consistent with human biomonitoring data showing that exposure to sulfonamide-based precursors appears to be minimal among non-occupationally exposed populations.

To our knowledge, the perfluoroether compounds NFDHA, PFMPA, PFMBA, and PFEESA have not previously been measured in humans. They have been detected in environmental media in North Carolina [85], China [86], and Australia [87]. In our study, these compounds were infrequently detected at low concentrations. The 95th percentile of NFDHA was considerably higher than the other perfluoroether compounds (0.171 µg/L), which could indicate potentially unique sources of exposure; however, NFDHA was under-recovered in spiked reference serum samples (median = 45.5% and 55.3% for low and high spikes), and these results should be interpreted with caution. Additional biomonitoring data on perfluoroether compounds are needed to corroborate these findings.

This study has several strengths, including the geographically diverse sample as well as the collection of multiple sociodemographic, lifestyle, and obstetrical history characteristics. By examining exposure to these compounds in a perimenopausal cohort of participants with obstetrical history data, we were able to examine the long-term impacts of pregnancy and lactation on midlife PFAS exposure. These findings will help inform risk assessment of novel exposures in potentially vulnerable life stages. In addition to providing novel Canadian biomonitoring data for some PFAS, we also provide the only Canadian biomonitoring data for PFAS around the time of the COVID-19 pandemic. Despite the geographically diverse sample of participants in the MIREC ENDO follow-up study, our sample size is relatively small, and these results may not be representative of the Canadian population. Few participants identified their race and ethnicity as being other than exclusively White, and similarly few participants were born outside of Canada. Although we identified some differences in PFAS concentrations according to these two factors, further research would be needed to specifically identify sub-populations that may be disproportionately impacted by exposure to PFAS. Finally, the presence of a chemical in blood does not necessarily mean that this chemical is associated with an adverse health effect. There is a dearth of evidence examining the potential health impacts of exposure to the novel PFAS measured in our study, especially at lower levels of exposure typically experienced by the general population. Further studies are needed to provide this evidence and should consider obstetrical history characteristics as potential confounders for health outcomes influenced by parity and breastfeeding.

## Conclusion

We report the first Canadian biomonitoring data for several alternative and precursor PFAS. The high degree of detection suggests that exposure to these PFAS may be widespread, though at low concentrations for some compounds; larger Canadian studies are needed to corroborate these findings. Our results support the hypothesis that pregnancy and breastfeeding are excretion pathways for legacy and alternative PFAS. These findings will guide future research investigating associations between concentrations of legacy, alternative, and precursor PFAS and health outcomes.

### Electronic supplementary material

Below is the link to the electronic supplementary material.

Supplementary Material 1

## Data Availability

The datasets generated and/or analysed during the current study are not publicly available to respect privacy, but individuals may apply to access the data through the MIREC Biobank (www.mirec-canada.ca/en/research).

## Abbreviations

MIREC: Maternal-infant research on environmental chemicals
LOD: Limit of detection
CAD: Canadian dollars
PFAS: Per- and polyfluoroalkyl substances

## References

[1] Buck RC, Franklin J, Berger U, Conder JM, Cousins IT, de Voogt P (2011). Perfluoroalkyl and polyfluoroalkyl substances in the environment: terminology, classification, and origins. Integr Environ Assess Manag.

[2] Panieri E, Baralic K, Djukic-Cosic D, Djordjevic AB, Saso L. PFAS Molecules: A Major Concern for the Human Health and the Environment. Toxics 2022, Vol 10, Page 44. 2022;10:44. 10.3390/toxics10020044 PMC8878656 35202231

[3] Health Canada. Human Biomonitoring of Environmental Chemicals In Canada: Results of the Canadian Health Measures Survey Cycle 6 (2018–2019). 2021.

[4] Kärrman A, Mueller JF, van Bavel B, Harden F, Toms L-ML, Lindström G (2006). Levels of 12 perfluorinated chemicals in pooled Australian serum, collected 2002–2003, in relation to age, gender, and region. Environ Sci Technol.

[5] Health Canada. Canadian Biomonitoring Dashboard [Internet]. 2023. https://health-infobase.canada.ca/biomonitoring/.

[6] Uhl M, Schoeters G, Govarts E, Bil W, Fletcher T, Haug LS et al. PFASs: what can we learn from the European Human Biomonitoring Initiative HBM4EU. Int J Hyg Environ Health. 2023;250. 10.1016/j.ijheh.2023.114168 37068413

[7] Centers for Disease Control and Prevention. National Report on Human exposure to Environmental Chemicals, Biomonitoring Data tables for Environmental Chemicals. Atlanta, Georgia.

[8] Trudel D, Horowitz L, Wormuth M, Scheringer M, Cousins IT, Hungerbühler K (2008). Estimating consumer exposure to PFOS and PFOA. Risk Anal.

[9] Fromme H, Tittlemier SA, Völkel W, Wilhelm M, Twardella D (2009). Perfluorinated compounds – exposure assessment for the general population in western countries. Int J Hyg Environ Health.

[10] Poothong S, Papadopoulou E, Padilla-Sánchez JA, Thomsen C, Haug LS. Multiple pathways of human exposure to poly- and perfluoroalkyl substances (PFASs): from external exposure to human blood. Environ Int. 2020;134. 10.1016/j.envint.2019.105244 31711019

[11] Sunderland E, Hu X, Dassuncao C, Tokranov A, Wagner C, Allen J (2019). A review of pathways of human exposure to poly- and perfluoroalkyl substances (PFASs) and present understanding of health effects. J Expo Sci Environ Epidemiol.

[12] Agency for Toxic Substances and Disease Registry (ATSDR). Toxicological profile for perfluoroalkyls. Atlanta, Georgia; 2021. 37220203

[13] Chiu WA, Lynch MT, Lay CR, Antezana A, Malek P, Sokolinski S (2022). Bayesian estimation of Human Population Toxicokinetics of PFOA, PFOS, PFHxS, and PFNA from studies of contaminated drinking Water. Environ Health Perspect.

[14] Costello E, Rock S, Stratakis N, Eckel SP, Walker DI, Valvi D et al. Exposure to per- and polyfluoroalkyl substances and markers of Liver Injury: a systematic review and Meta-analysis. Environ Health Perspect. 2022;130. 10.1289/EHP10092 PMC9044977 35475652

[15] Fenton SE, Ducatman A, Boobis A, DeWitt JC, Lau C, Ng C, et al. Per- and Polyfluoroalkyl Substance Toxicity and Human Health Review: current state of knowledge and strategies for informing Future Research. Environ Toxicol Chem. Wiley Blackwell; 2021. pp. 606–30. 10.1002/etc.4890 PMC7906952 33017053

[16] Ho SH, Soh SXH, Wang MX, Ong J, Seah A, Wong Y (2022). Perfluoroalkyl substances and lipid concentrations in the blood: a systematic review of epidemiological studies. Sci Total Environ.

[17] US EPA. IRIS Toxicological Review of Perfluorobutanoic Acid (PFBA, CASRN 375-22-4) and Related Salts [Internet]. 2022 Dec. https://iris.epa.gov/static/pdfs/0701tr.pdf. 38324661

[18] US EPA. IRIS Toxicological Review of Perfluorohexanoic Acid [PFHxA, CASRN 307-24-4] and Related Salts [Internet]. 2023 Apr. https://iris.epa.gov/static/pdfs/0704tr.pdf. 38484103

[19] US EPA. IRIS Toxicological Review of Perfluorohexanesulfonic Acid (PFHxS) and Related Salts (Public Comment and External Review Draft) [Internet]. Washington DC; 2023. Available from: https://iris.epa.gov/Document/&deid=355410

[20] US EPA. IRIS Toxicological Review of Perfluorononanoic Acid (PFNA) and Related Salts (Public Comment and External Review Draft) [Internet]. Washington DC; 2023. Available from: https://iris.epa.gov/Document/&deid=355409

[21] US EPA. IRIS Toxicological Review of Perfluorodecanoic Acid (PFDA) and Related Salts (Public Comment and External Review Draft) [Internet]. Washington DC; 2023. Available from: https://iris.epa.gov/Document/&deid=354408 39836792

[22] Organisation for Economic Co-operation and Development. Toward a new comprehensive global database of per- and polyfluoroalkyl substances (PFASs). Paris, France; 2018.

[23] Bil W, Zeilmaker MJ, Bokkers BGH. Internal relative potency factors for the Risk Assessment of mixtures of Per- and polyfluoroalkyl substances (PFAS) in human biomonitoring. Environ Health Perspect. 2022;130. 10.1289/EHP10009 PMC9320915 35881550

[24] Brendel S, Fetter É, Staude C, Vierke L, Biegel-Engler A. Short-chain perfluoroalkyl acids: environmental concerns and a regulatory strategy under REACH. Environ Sci Eur. 2018;30. 10.1186/s12302-018-0134-4 PMC5834591 29527446

[25] Brennan NM, Evans AT, Fritz MK, Peak SA, von Holst HE. Trends in the regulation of per-and polyfluoroalkyl substances (PFAS): a scoping review. Int J Environ Res Public Health. 2021;18. 10.3390/ijerph182010900 PMC8536021 34682663

[26] Government of Canada. Regulations Amending the Prohibition of Certain Toxic Substances Regulations. 2012. Canada Gazette [Internet]. 2016 [cited 2023 Oct 29];150. https://gazette.gc.ca/rp-pr/p2/2016/2016-10-05/html/sor-dors252-eng.html.

[27] Pollock T, Karthikeyan S, Walker M, Werry K, St-Amand A (2021). Trends in environmental chemical concentrations in the Canadian population: Biomonitoring data from the Canadian Health measures Survey 2007–2017. Environ Int.

[28] Calafat AM, Wong L-Y, Kuklenyik Z, Reidy JA, Needham LL (2007). Polyfluoroalkyl Chemicals in the U.S. Population: data from the National Health and Nutrition Examination Survey (NHANES) 2003–2004 and comparisons with NHANES 1999–2000. Environ Health Perspect.

[29] Kato K, Wong L-Y, Jia LT, Kuklenyik Z, Calafat AM (2011). Trends in exposure to Polyfluoroalkyl Chemicals in the U.S. Population: 1999 – 2008. Environ Sci Technol.

[30] Olsen GW, Lange CC, Ellefson ME, Mair DC, Church TR, Goldberg CL (2012). Temporal trends of perfluoroalkyl concentrations in American Red Cross adult blood donors, 2000–2010. Environ Sci Technol.

[31] Liu Y, Pereira AS, Beesoon S, Vestergren R, Berger U, Olsen GW (2015). Temporal trends of perfluorooctanesulfonate isomer and enantiomer patterns in archived Swedish and American serum samples. Environ Int.

[32] Schoeters G, Govarts E, Bruckers L, Den Hond E, Nelen V, De Henauw S (2017). Three cycles of human biomonitoring in Flanders – Time trends observed in the flemish environment and health study. Int J Hyg Environ Health.

[33] Shirke A V., Radke EG, Lin C, Blain R, Vetter N, Lemeris C, et al. Expanded Systematic Evidence Map for Hundreds of Per- and Polyfluoroalkyl Substances (PFAS) and Comprehensive PFAS Human Health Dashboard. Environ Health Perspect [Internet]. 2024 [cited 2024 Mar 12];132:26001. Available from: https://ehp.niehs.nih.gov/doi/10.1289/EHP13423 10.1289/EHP13423 PMC10846678 38319881

[34] Lalonde B, Garron C (2022). Perfluoroalkyl substances (PFASs) in the Canadian freshwater environment. Arch Environ Contam Toxicol.

[35] Arbuckle TE, Fraser WD, Fisher M, Davis K, Liang CL, Lupien N (2013). Cohort Profile: the Maternal-Infant Research on Environmental Chemicals Research Platform. Paediatr Perinat Epidemiol.

[36] Fisher M, Muckle G, Lanphear B, Arbuckle TE, Braun JM, Zidek A (2023). Cohort Profile Update: the Canadian Maternal-Infant Research on Environmental Chemicals Child Development Study (MIREC- CD -PLUS). Paediatr Perinat Epidemiol.

[37] Statistics Canada. Tables 13-10-0373-01 Overweight and obesity based on measured body mass index, by age group and sex. 2021.

[38] Hornung R, Reed L (1990). Estimation of average concentration in the Presence of nondetectable values. Appl Occup Environ Hyg.

[39] VanNoy BN, Lam J, Zota AR (2018). Breastfeeding as a predictor of serum concentrations of per- and Polyfluorinated Alkyl substances in Reproductive-aged women and young children: a Rapid systematic review. Curr Environ Health Rep.

[40] Blomberg AJ, Norén E, Haug LS, Lindh C, Sabaredzovic A, Pineda D et al. Estimated transfer of Perfluoroalkyl Substances (PFAS) from maternal serum to breast milk in women highly exposed from contaminated drinking water: a study in the Ronneby Mother-Child Cohort. Environ Health Perspect. 2023;131. 10.1289/EHP11292 PMC9869870 36688826

[41] Liu J, Gao X, Wang Y, Leng J, Li J, Zhao Y (2021). Profiling of emerging and legacy per-/polyfluoroalkyl substances in serum among pregnant women in China. Environ Pollut.

[42] Kim K, Bennett D, Calafat A, Hertz-Picciotto I, Shin H. Temporal trends and determinants of serum concentrations of per- and polyfluoroalkyl substances among Northern California mothers with a young child, 2009–2016. Environ Res. 2020;186. 10.1016/j.envres.2020.109491 PMC7363519 32361076

[43] Bach CC, Matthiesen NB, Olsen J, Henriksen TB. Conditioning on Parity in Studies of Perfluoroalkyl Acids and Time to Pregnancy: An Example from the Danish National Birth Cohort. Environ Health Perspect. 2018;126. 10.1289/EHP1493 PMC6371644 30417653

[44] Appel M, Forsthuber M, Ramos R, Widhalm R, Granitzer S, Uhl M et al. The transplacental transfer efficiency of per- and polyfluoroalkyl substances (PFAS): a first meta-analysis. J Toxicol Environ Health B Crit Rev. Taylor and Francis Ltd.; 2022. pp. 23–42. 10.1080/10937404.2021.2009946 34930098

[45] Forsthuber M, Kaiser AM, Granitzer S, Hassl I, Hengstschläger M, Stangl H (2020). Albumin is the major carrier protein for PFOS, PFOA, PFHxS, PFNA and PFDA in human plasma. Environ Int.

[46] Rawn DFK, Dufresne G, Clément G, Fraser WD, Arbuckle TE. Perfluorinated alkyl substances in Canadian human milk as part of the Maternal-Infant Research on Environmental Chemicals (MIREC) study. Science of the Total Environment. 2022;831. 10.1016/j.scitotenv.2022.154888 35367260

[47] Bach CC, Matthiesen NB, Olsen J, Henriksen TB. Conditioning on Parity in Studies of Perfluoroalkyl Acids and Time to Pregnancy: An Example from the Danish National Birth Cohort. Environ Health Perspect [Internet]. 2018 [cited 2023 May 16];126. Available from: https://ehp.niehs.nih.gov/doi/10.1289/EHP1493 10.1289/EHP1493 PMC6371644 30417653

[48] US CDC. National Report on Human exposure to Environmental Chemicals. Perfluoroalkyl and Polyfluoroalkyl Substances: Surfactants.

[49] Health Canada. Canadian Biomonitoring Dashboard [Internet]. 2023. Available from: https://health-infobase.canada.ca/biomonitoring/.

[50] Rawn DFK, Dufresne G, Clément G, Fraser WD, Arbuckle TE. Perfluorinated alkyl substances in Canadian human milk as part of the Maternal-Infant Research on Environmental Chemicals (MIREC) study. Science of the Total Environment. 2022;831. 10.1016/j.scitotenv.2022.154888 35367260

[51] Zheng G, Eick SM, Salamova A. Elevated levels of Ultrashort- and short-chain perfluoroalkyl acids in US homes and people. Environ Sci Technol. 2023. 10.1021/acs.est.2c06715 PMC10603771 37818968

[52] Göckener B, Weber T, Rüdel H, Bücking M, Kolossa-Gehring M (2020). Human biomonitoring of per- and polyfluoroalkyl substances in German blood plasma samples from 1982 to 2019. Environ Int.

[53] Wang Z, Cousins IT, Scheringer M, Hungerbühler K (2013). Fluorinated alternatives to long-chain perfluoroalkyl carboxylic acids (PFCAs), perfluoroalkane sulfonic acids (PFSAs) and their potential precursors. Environ Int.

[54] Wang S, Huang J, Yang Y, Hui Y, Ge Y, Larssen T (2013). First report of a Chinese PFOS alternative overlooked for 30 years: its toxicity, persistence, and presence in the environment. Environ Sci Technol.

[55] Munoz G, Liu J, Vo Duy S, Sauvé S (2019). Analysis of F-53B, Gen-X, ADONA, and emerging fluoroalkylether substances in environmental and biomonitoring samples: a review. Trends Environ Anal Chem.

[56] Sun M, Arevalo E, Strynar M, Lindstrom A, Richardson M, Kearns B (2016). Legacy and emerging perfluoroalkyl substances are important drinking Water contaminants in the Cape Fear River Watershed of North Carolina. Environ Sci Technol Lett.

[57] Göckener B, Weber T, Rüdel H, Bücking M, Kolossa-Gehring M. Human biomonitoring of per- and polyfluoroalkyl substances in German blood plasma samples from 1982 to 2019. Environ Int. 2020;145:106123. 10.1016/j.envint.2020.106123 32949877

[58] Kotlarz N, McCord J, Collier D, Suzanne Lea C, Strynar M, Lindstrom AB (2020). Measurement of novel, drinking water-associated pfas in blood from adults and children in Wilmington, North Carolina. Environ Health Perspect.

[59] Fromme H, Fuchs V, Albrecht M, Aschenbrenner B, Röhl C, Janitzki N et al. Polychlorinated dioxins and dibenzofurans (PCDD/F), polybrominated dioxins and dibenzofurans (PBDD/F), polychlorinated biphenyls (PCB), polybrominated diphenyl ethers (PBDE), and per- and polyfluoroalkyl substances (PFAS) in German breast milk samples (LUPE 8). Sci Total Environ. 2022;825. 10.1016/j.scitotenv.2022.154066 35217048

[60] Fromme H, Wöckner M, Roscher E, Völkel W (2017). ADONA and perfluoroalkylated substances in plasma samples of German blood donors living in South Germany. Int J Hyg Environ Health.

[61] Ruan T, Lin Y, Wang T, Liu R, Jiang G (2015). Identification of novel polyfluorinated ether sulfonates as PFOS alternatives in municipal sewage sludge in China. Environ Sci Technol.

[62] Duan Y, Sun H, Yao Y, Meng Y, Li Y (2020). Distribution of novel and legacy per-/polyfluoroalkyl substances in serum and its associations with two glycemic biomarkers among Chinese adult men and women with normal blood glucose levels. Environ Int.

[63] Jin H, Lin S, Dai W, Feng L, Li T, Lou J (2020). Exposure sources of perfluoroalkyl acids and influence of age and gender on concentrations of chlorinated polyfluorinated ether sulfonates in human serum from China. Environ Int.

[64] Pan Y, Zhu Y, Zheng T, Cui Q, Buka SL, Zhang B (2017). Novel Chlorinated Polyfluorinated Ether sulfonates and Legacy Per-/Polyfluoroalkyl substances: placental transfer and relationship with serum albumin and glomerular filtration rate. Environ Sci Technol.

[65] Shi Y, Vestergren R, Xu L, Zhou Z, Li C, Liang Y (2016). Human exposure and Elimination Kinetics of Chlorinated Polyfluoroalkyl Ether Sulfonic acids (Cl-PFESAs). Environ Sci Technol.

[66] Han F, Wang Y, Li J, Lyu B, Liu J, Zhang J (2023). Occurrences of legacy and emerging per- and polyfluoroalkyl substances in human milk in China: results of the third National Human milk survey (2017–2020). J Hazard Mater.

[67] Awad R, Zhou Y, Nyberg E, Namazkar S, Yongning W, Xiao Q (2020). Emerging per- and polyfluoroalkyl substances (PFAS) in human milk from Sweden and China. Environ Sci Process Impacts.

[68] Yao J, Dong Z, Jiang L, Pan Y, Zhao M, Bai X (2023). Emerging and Legacy Perfluoroalkyl substances in Breastfed Chinese infants: renal clearance, body burden, and implications. Environ Health Perspect.

[69] Zheng P, Liu Y, An Q, Yang X, Yin S, Ma LQ (2022). Prenatal and postnatal exposure to emerging and legacy per-/polyfluoroalkyl substances: levels and transfer in maternal serum, cord serum, and breast milk. Sci Total Environ.

[70] Nyström J, Benskin JP, Plassmann M, Sandblom O, Glynn A, Lampa E (2022). Demographic, life-style and physiological determinants of serum per- and polyfluoroalkyl substance (PFAS) concentrations in a national cross-sectional survey of Swedish adolescents. Environ Res.

[71] Brase RA, Mullin EJ, Spink DC (2021). Legacy and emerging per-and polyfluoroalkyl substances: Analytical techniques, environmental fate, and health effects. Int J Mol Sci.

[72] Liu Y, Richardson ES, Derocher AE, Lunn NJ, Lehmler HJ, Li X (2018). Hundreds of unrecognized Halogenated contaminants discovered in Polar Bear serum. Angew Chem Int Ed.

[73] Gebbink WA, Bossi R, Rigét FF, Rosing-Asvid A, Sonne C, Dietz R (2016). Observation of emerging per- and polyfluoroalkyl substances (PFASs) in Greenland Marine mammals. Chemosphere.

[74] Spaan KM, Van Noordenburg C, Plassmann MM, Schultes L, Shaw S, Berger M (2020). Fluorine Mass Balance and Suspect Screening in Marine mammals from the Northern Hemisphere. Environ Sci Technol.

[75] Gannon SA, Fasano WJ, Mawn MP, Nabb DL, Buck RC, Buxton LW (2016). Absorption, distribution, metabolism, excretion, and kinetics of 2,3,3,3-tetrafluoro-2-(heptafluoropropoxy)propanoic acid ammonium salt following a single dose in rat, mouse, and cynomolgus monkey. Toxicology.

[76] Thompson CM, Fitch SE, Ring C, Rish W, Cullen JM, Haws LC (2019). Development of an oral reference dose for the perfluorinated compound GenX. J Appl Toxicol.

[77] EFSA. Scientific opinion on the safety evaluation of the substance, 3H-perfluoro-3-[(3-methoxy-propoxy)propanoic acid], ammonium salt, CAS 958445-44-8, for use in food contact materials. EFSA J. 2011;9.

[78] US Centers for Disease Control. Fourth National Report on Human Exposure to Environmental Chemicals. 2019.

[79] UNEP. Technical paper on the identification and assessment of alternatives to the use of perfluorooctane sulfonic acid in open applications. 2012.

[80] Wang Y, Chang W, Wang L, Zhang Y, Zhang Y, Wang M (2019). A review of sources, multimedia distribution and health risks of novel fluorinated alternatives. Ecotoxicol Environ Saf.

[81] Poothong S, Thomsen C, Padilla-Sanchez JA, Papadopoulou E, Haug LS (2017). Distribution of Novel and well-known poly- and perfluoroalkyl substances (PFASs) in human serum, plasma, and whole blood. Environ Sci Technol.

[82] Göckener B, Fliedner A, Rüdel H, Fettig I, Koschorreck J (2021). Exploring unknown per- and polyfluoroalkyl substances in the German environment – the total oxidizable precursor assay as helpful tool in research and regulation. Sci Total Environ.

[83] Mejia Avendaño S, Liu J (2015). Production of PFOS from aerobic soil biotransformation of two perfluoroalkyl sulfonamide derivatives. Chemosphere.

[84] Plumlee MH, Mcneill K, Reinhard M (2009). Indirect photolysis of perfluorochemicals: Hydroxyl radical-initiated oxidation of N-ethyl perfluorooctane sulfonamido acetate (N-EtFOSAA) and other perfluoroalkanesulfonamides. Environ Sci Technol.

[85] Shimizu MS, Mott R, Potter A, Zhou J, Baumann K, Surratt JD (2021). Atmospheric deposition and annual flux of legacy perfluoroalkyl substances and replacement perfluoroalkyl ether carboxylic acids in Wilmington, NC, USA. Environ Sci Technol Lett.

[86] Liu Z, Zhou J, Xu Y, Lu J, Chen J, Wang J (2022). Distributions and sources of traditional and emerging per- and polyfluoroalkyl substances among multiple environmental media in the Qiantang River watershed, China. RSC Adv.

[87] Alghamdi W, Marchiandi J, Szabo D, Samandra S, Clarke BO (2022). Release of per- and polyfluoroalkyl substances (PFAS) from a waste management facility fire to an urban creek. J Hazard Mater Adv.

