# Supplementary material for: Serum concentrations of legacy, alternative, and precursor per- and polyfluoroalkyl substances: a descriptive analysis of adult female participants in the MIREC-ENDO study
Source: Environ Health. 2024 Jun 10;23:55. doi: 10.1186/s12940-024-01085-z (PMC11163811; doi:10.1186/s12940-024-01085-z)

Supplemental Table 1 – Per- and polyfluoroalkyl substances analyzed in the MIREC-ENDO study (2018 – 2021)

| ***Abbreviation*** | ***Name - Acid Form*** | ***CAS#*** |
| --- | --- | --- |
| Perfluorinated carboxylic acids | | |
| PFBA | Perfluorobutanoic acid | 375-22-4 |
| PFPeA | Perfluoropentanoic acid | 2706-90-3 |
| PFHxA | Perfluorohexanoic acid | 307-24-4 |
| PFHpA | Perfluoroheptanoic acid | 375-85-9 |
| PFOA | Perfluorooctanoic acid | 335-67-1 |
| PFNA | Perfluorononanoic acid | 375-95-1 |
| PFDA | Perfluorodecanoic acid | 335-76-2 |
| PFUDA | Perfluoroundecanoic acid | 2058-94-8 |
| PFDoA | Perfluorododecanoic acid | 307-55-1 |
| PFTrDA | Perfluorotridecanoic acid | 72629-94-8 |
| PFTeDA | Perfluorotetradecanoic acid | 376-06-7 |
| Perfluorinated sulfonic acids | | |
| PFBS | Perfluorobutanesulfonate | 375-73-5 |
| PFPeS | Perfluoropentanesulfonate | 2706-91-4 |
| PFHxS | Perfluorohexanesulfonate | 355-46-4 |
| PFHpS | Perfluoroheptanesulfonate | 375-92-8 |
| PFOS | Perfluorooctanesulfonate | 1763-23-1 |
| PFNS | Perfluorononanesulfonate | 68259-12-1 |
| PFDS | Perfluorodecanesulfonate | 335-77-3 |
| PFDoS | Perfluorododecanesulfonate | 79780-39-5 |
| Fluorotelomer sulfonic acids | | |
| 4:2 FTS | 4:2 fluorotelomersulfonate | 757124-72-4 |
| 6:2 FTS | 6:2 fluorotelomersulfonate | 27619-97-2 |
| 8:2 FTS | 8:2 fluorotelomersulfonate | 39108-34-4 |
| Fluorotelomer carboxylic acids | | |
| 3:3 FTCA | 3:3 perfluorohexanoic acid | 356-02-5 |
| 5:3 FTCA | 5:3 perfluorooctanoic acid | 914637-49-3 |
| 7:3 FTCA | 7:3 perfluorodecanoic acid | 812-70-4 |
| Perfluorooctane sulfonamides | | |
| PFOSA | Perfluorooctanesulfonamide | 754-91-6 |
| N-MeFOSA | N-Methylperfluorooctanesulfonamide | 31506-32-8 |
| N-EtFOSA | N-Ethylperfluorooctanesulfonamide | 4151-50-2 |
| Perfluorooctane sulfonamidoacetic acids | | |
| N-MeFOSAA | N-Methylperfluorooctanesulfonamidoacetic acid | 2355-31-9 |
| N-EtFOSAA | N-Ethylperfluorooctanesulfonamidoacetic acid | 2991-50-6 |
| Perfluorooctane sulfonamide ethanols | | |
| N-MeFOSE | N-Methylperfluorooctanesulfonamidoethanol | 24448-09-7 |
| N-EtFOSE | N-Ethylperfluorooctanesulfonamidoethanol | 1691-99-2 |
| Per- and polyfluoroether carboxylic acids | | |
| HFPO-DA (GenX) | 2,3,3,3-Tetrafluoro-2-(1,1,2,2,3,3,3-heptafluoropropoxy)propanoic acid | 13252-13-6 |
| ADONA | Dodecafluoro-3H-4,8-dioxanonanoic acid | 919005-14-4 |
| NFDHA | Perfluoro-3,6-dioxaheptanoic acid | 151772-58-6 |
| PFMPA | Perfluoro-3-methoxypropanoic acid | 377-73-1 |
| PFMBA | Perfluoro-4-methoxybutanoic acid | 863090-89-5 |
| Per- and polyfluoroether sulfonic acids | | |
| 9Cl-PF3ONS | 9-chlorohexadecafluoro-3-oxanonane-1-sulfonic acid | 756426-58-1 |
| 11Cl-PF3OUdS | 11-chloroeicosafluoro-3-oxaundecane-1-sulfonic acid | 763051-92-9 |
| PFEESA | Perfluoro(2-ethoxyethane)sulfonic acid | 113507-82-7 |

Supplemental Table 2 – Median and interquartile range limits of detection (µg/L) for batch-specific blank samples (15 batches) (MIREC-ENDO, 2018 – 2021)

|  | 25^th^ percentile | Median | 75^th^ percentile |
| --- | --- | --- | --- |
| Perfluoroalkyl carboxylates | | | |
| PFBA | 0.089 | 0.099 | 0.132 |
| PFPeA | 0.039 | 0.042 | 0.054 |
| PFHxA | 0.053 | 0.062 | 0.074 |
| PFHpA | 0.040 | 0.049 | 0.056 |
| PFOA | 0.171 | 0.190 | 0.209 |
| PFNA | 0.059 | 0.071 | 0.076 |
| PFDA | 0.074 | 0.083 | 0.099 |
| PFUDA | 0.059 | 0.077 | 0.093 |
| PFDoA | 0.058 | 0.070 | 0.076 |
| PFTrDA | 0.080 | 0.106 | 0.112 |
| PFTeDA | 0.135 | 0.155 | 0.174 |
| Perfluoroalkyl sulfonates | | | |
| PFBS | 0.001 | 0.003 | 0.004 |
| PFPeS | 0.002 | 0.003 | 0.003 |
| PFHxS | 0.003 | 0.003 | 0.005 |
| PFHpS | 0.002 | 0.002 | 0.003 |
| PFOS | 0.004 | 0.005 | 0.012 |
| PFNS | 0.001 | 0.002 | 0.003 |
| PFDS | 0.001 | 0.002 | 0.003 |
| PFDoS | 0.002 | 0.002 | 0.003 |
| Fluorotelomer sulfonates | | | |
| 4:2 FTS | 0.004 | 0.005 | 0.007 |
| 6:2 FTS | 0.010 | 0.015 | 0.026 |
| 8:2 FTS | 0.007 | 0.009 | 0.011 |
| Fluorotelomer carboxylates | | | |
| 3:3 FTCA | 0.023 | 0.031 | 0.04 |
| 5:3 FTCA | 0.025 | 0.046 | 0.086 |
| 7:3 FTCA | 0.041 | 0.078 | 0.109 |
| Perfluorooctane sulfonamides | | | |
| PFOSA | 0.001 | 0.002 | 0.002 |
| N-MeFOSA | 0.005 | 0.006 | 0.009 |
| N-EtFOSA | 0.007 | 0.008 | 0.009 |
| Perfluorooctane sulfonamidoacetic acids | | | |
| N-MeFOSAA | 0.006 | 0.008 | 0.008 |
| N-EtFOSAA | 0.006 | 0.009 | 0.012 |
| Perfluorooctane sulfonamide ethanols | | | |
| N-MeFOSE | 0.014 | 0.017 | 0.020 |
| N-EtFOSE | 0.015 | 0.020 | 0.023 |
| Per- and polyfluoroether carboxylates | | | |
| HFPO-DA | 0.003 | 0.003 | 0.006 |
| ADONA | 0.001 | 0.001 | 0.001 |
| NFDHA | 0.049 | 0.061 | 0.08 |
| PFMPA | 0.006 | 0.007 | 0.008 |
| PFMBA | 0.002 | 0.002 | 0.002 |
| Ether sulfonates | | | |
| 9Cl-PF3ONS | 0.002 | 0.005 | 0.007 |
| 11Cl-PF3OUdS | 0.003 | 0.005 | 0.008 |
| PFEESA | 0.001 | 0.001 | 0.002 |

Supplemental Table 3 – Percent recovery of spiked surrogate standards in blank samples from 15 batches in the MIREC-ENDO follow-up study (2018 – 2021)

| Surrogate standards | Corresponding native compounds | Surrogate spike concentration (µg/L) | Percent recovery | | |
| --- | --- | --- | --- | --- | --- |
|  |  |  | Min | Median | Max |
| ^13^C_2_-PFTeDA | PFTeDA | 5 | 58.3 | 80.5 | 102 |
| ^13^C_9_-PFNA | PFNA | 5 | 52.7 | 80.9 | 94 |
| D_5_-N-EtFOSAA | N-EtFOSAA | 20 | 53.4 | 83.4 | 114 |
| ^13^C_2_-PFTeA and ^13^C_2_- PFDoA^1^ | PFTrDA | - | 58.7 | 83.6 | 102 |
| D_3_-N-MeFOSAA | N-MeFOSAA | 20 | 54.5 | 83.7 | 106 |
| ^13^C2-PFDoA | PFDoA | 5 | 59.1 | 86.5 | 103 |
| ^13^C_3_-HFPO-DA | ADONA | 40 | 56.6 | 87.4 | 106 |
|  | 11Cl-PF3OUdS |  |  |  |  |
|  | HFPO-DA |  |  |  |  |
|  | 9Cl-PF3ONS |  |  |  |  |
| ^13^C_2_-8:2 FTS | 8:2 FTS | 20 | 60.7 | 90.3 | 108 |
| ^13^C_2_-6:2 FTS | 6:2 FTS | 20 | 58.4 | 91.7 | 104 |
| ^13^C_3_-PFHxS | PFHxS | 10 | 57.4 | 91.8 | 109 |
|  | PFPeS |  |  |  |  |
| ^13^C_6_-PFDA | PFDA | 5 | 60 | 91.8 | 111 |
| ^13^C_4_-PFBA | PFBA | 40 | 64 | 92.1 | 104 |
| ^13^C_5_-PFPeA | PFMBA | 20 | 61.9 | 92.3 | 105 |
|  | PFMPA |  |  |  |  |
|  | PFPeA |  |  |  |  |
|  | 3:3 FTCA |  |  |  |  |
| ^13^C_8_-PFOS | PFDoS | 10.1 | 60.6 | 94.2 | 107 |
|  | PFDS |  |  |  |  |
|  | PFHpS |  |  |  |  |
|  | PFNS |  |  |  |  |
|  | PFOS |  |  |  |  |
| ^13^C_8_-PFOA | PFOA | 10 | 59.4 | 94.9 | 115 |
| ^13^C_7_-PFUDA | PFUDA | 5 | 62.6 | 96.4 | 113 |
| ^13^C_5_-PFHxA | 5:3 FTCA | 10 | 63.9 | 97.8 | 118 |
|  | NFDHA |  |  |  |  |
|  | PFEESA |  |  |  |  |
|  | PFHxA |  |  |  |  |
|  | 7:3 FTCA |  |  |  |  |
| ^13^C_3_-PFBS | PFBS | 10 | 61.3 | 98.4 | 120 |
| D_5_-N-EtFOSA | N-EtFOSA | 10 | 65.5 | 101 | 111 |
| ^13^C_4_-PFHpA | PFHpA | 10 | 59 | 102 | 130 |
| D_3_-N-MeFOSA | N-MeFOSA | 10 | 71.7 | 107 | 121 |
| ^13^C_8_-PFOSA | PFOSA | 10 | 70.5 | 108 | 122 |
| D_9_-N-EtFOSE | N-EtFOSE | 100 | 71.7 | 111 | 134 |
| ^13^C_2_-4:2 FTS | 4:2 FTS | 20.2 | 76.5 | 113 | 137 |
| D_7_-N-MeFOSE | N-MeFOSE | 100 | 98.9 | 149 | 172 |

^1^ Calculated as the average of ^13^C_2_-PFTeA and ^13^C_2_- PFDoA

Supplemental Table 4 – Percent recovery of native per- and polyfluoroalkyl substances from two spiked reference serum samples from 15 batches in the MIREC-ENDO follow-up study (2018 – 2021)

|  | Low spike concentration (µg/L) | Percent recovery | | | Inter-  assay CV (%) | High spike concentration (µg/L) | Percent recovery | | | Inter-  assay CV (%) |
| --- | --- | --- | --- | --- | --- | --- | --- | --- | --- | --- |
|  |  | Min | Median | Max |  |  | Min | Median | Max |  |
| 3:3 FTCA | 6.4 | 22.3 | 27.5 | 36.9 | 14.4 | 20 | 18.6 | 23.3 | 27.7 | 9.7 |
| NFDHA | 3.2 | 20.3 | 45.5 | 113 | 48.0 | 10 | 30.5 | 55.3 | 86.9 | 33.5 |
| N-MeFOSE | 16 | 70.1 | 74.5 | 81.8 | 4.1 | 50 | 70.0 | 74.2 | 77.5 | 3.5 |
| PFOS | 2.3 | 74.9 | 80.3 | 84.7 | 3.4 | 8.65 | 96.8 | 102 | 111 | 3.6 |
| PFDoS | 1.6 | 80.2 | 85.8 | 92.8 | 5.3 | 5.01 | 76.2 | 81.1 | 86.7 | 3.7 |
| PFEESA | 1.6 | 79.2 | 89.7 | 100 | 6.2 | 5 | 78.0 | 87.5 | 108 | 9.0 |
| 7:3 FTCA | 40 | 82.6 | 91.9 | 104 | 5.9 | 125 | 84.1 | 95.7 | 101 | 5.1 |
| PFMBA | 1.6 | 86.2 | 92.0 | 102 | 5.7 | 5 | 86.5 | 95.1 | 99.8 | 3.9 |
| PFMPA | 3.2 | 83.3 | 92.4 | 98.7 | 4.8 | 10 | 88.6 | 95.0 | 101 | 4.0 |
| PFHpA | 1.6 | 82.6 | 93.8 | 97.4 | 5.3 | 5 | 85.5 | 92.9 | 101 | 4.1 |
| PFOA | 1.6 | 84.8 | 94.0 | 104 | 5.7 | 6.5 | 82.6 | 88.8 | 93.1 | 3.7 |
| N-MeFOSA | 1.84 | 90.7 | 94.5 | 121 | 8.5 | 5.75 | 96.3 | 102 | 117 | 5.0 |
| PFBS | 1.6 | 84.2 | 95.0 | 126 | 10.8 | 5 | 82.4 | 89.2 | 106 | 7.5 |
| 5:3 FTCA | 40 | 81.6 | 95.1 | 111 | 6.8 | 125 | 80.7 | 95.3 | 105 | 7.1 |
| PFHpS | 1.6 | 88.4 | 95.4 | 104 | 4.1 | 5.01 | 93.3 | 98.5 | 107 | 3.6 |
| N-EtFOSA | 4 | 91.3 | 95.9 | 112 | 5.8 | 12.5 | 87.2 | 99.4 | 105 | 4.8 |
| PFOSA | 1.6 | 90.5 | 96.8 | 101 | 3.2 | 5 | 91.1 | 97.4 | 102 | 3.0 |
| 11Cl-PF3OUdS | 6.41 | 91.9 | 96.9 | 112 | 6.6 | 20 | 85.2 | 98.6 | 112 | 8.3 |
| PFTrDA | 1.6 | 89.5 | 98.6 | 107 | 4.2 | 5 | 94.6 | 102 | 113 | 4.2 |
| N-EtFOSAA | 1.6 | 84.3 | 98.6 | 112 | 8.0 | 5 | 90.8 | 105 | 116 | 6.9 |
| 4:2 FTS | 6.4 | 75.8 | 99.3 | 115 | 11.7 | 20 | 90.4 | 102 | 127 | 9.6 |
| PFBA | 6.4 | 96.2 | 99.5 | 106 | 2.9 | 20 | 95.7 | 99.4 | 103 | 2.2 |
| PFHxS | 1.6 | 94.9 | 100 | 110 | 3.5 | 6.5 | 90.4 | 100 | 104 | 3.2 |
| HFPO-DA | 6.08 | 90.0 | 101 | 123 | 8.5 | 19 | 73.4 | 106 | 116 | 12.4 |
| PFDS | 1.6 | 90.3 | 101 | 105 | 4.7 | 5 | 93.6 | 99.7 | 106 | 4.2 |
| PFNA | 1.6 | 97.5 | 101 | 112 | 4.7 | 5.64 | 95.4 | 100 | 105 | 2.8 |
| PFDoA | 1.6 | 93.9 | 102 | 109 | 4.3 | 5 | 91.7 | 102 | 106 | 3.9 |
| ADONA | 6.42 | 94.3 | 102 | 119 | 6.9 | 20.1 | 80.7 | 106 | 117 | 9.1 |
| PFHxA | 1.6 | 88.4 | 102 | 113 | 6.9 | 5 | 83.2 | 101 | 114 | 8.1 |
| PFPeA | 3.2 | 91.2 | 102 | 113 | 5.5 | 10 | 98.8 | 103 | 110 | 3.3 |
| PFNS | 1.6 | 92.8 | 102 | 105 | 4.4 | 5.01 | 94.4 | 99.6 | 104 | 2.9 |
| 9Cl-PF3ONS | 6.41 | 94.8 | 103 | 119 | 7.7 | 20 | 91.0 | 105 | 122 | 8.6 |
| PFPeS | 1.6 | 95.7 | 103 | 107 | 3.9 | 5.01 | 93.3 | 101 | 106 | 3.2 |
| 8:2 FTS | 6.4 | 95.0 | 105 | 122 | 7.1 | 20 | 98.9 | 109 | 114 | 3.7 |
| N-MeFOSAA | 1.6 | 84.1 | 106 | 121 | 9.8 | 5 | 97.7 | 113 | 124 | 8.2 |
| 6:2 FTS | 5.77 | 96.2 | 107 | 159 | 13.5 | 18 | 97.6 | 108 | 129 | 7.7 |
| PFTeDA | 1.6 | 98.4 | 107 | 124 | 6.4 | 5 | 98.3 | 106 | 113 | 4.0 |
| PFDA | 1.6 | 100 | 109 | 124 | 5.7 | 5 | 104 | 108 | 116 | 3.7 |
| PFUDA | 1.6 | 98.5 | 111 | 118 | 5.2 | 5 | 101 | 108 | 115 | 3.7 |
| N-EtFOSE | 12 | 105 | 113 | 121 | 3.8 | 37.5 | 109 | 124 | 131 | 5.8 |

Supplemental table 5 –Geometric means (95% CI) derived using maximum likelihood estimation for per- and polyfluoroalkyl substances (µg/L) with >50% detection among adult females from the MIREC-ENDO study (2018 – 2021)

|  | n | % > LOD^1^ | Geometric mean (95% CI) |
| --- | --- | --- | --- |
| PFOS | 289 | 100 | 1.669 (1.558, 1.788) |
| PFHxS | 289 | 100 | 0.315 (0.290, 0.343) |
| N-EtFOSE | 289 | 99.7 | 0.003 (0.002, 0.003) |
| PFNA | 289 | 99.3 | 0.362 (0.338, 0.389) |
| PFOSA | 289 | 99.0 | 0.002 (0.002, 0.002) |
| PFOA | 289 | 97.6 | 0.496 (0.429, 0.573) |
| 7:3 FTCA | 289 | 97.2 | 0.023 (0.018, 0.030) |
| 6:2 FTS | 269 | 85.1 | 0.011 (0.009, 0.015) |
| PFPeA | 289 | 79.6 | 0.005 (0.004, 0.007) |
| PFDA | 289 | 75.4 | 0.125 (0.113, 0.138) |
| PFBA | 289 | 71.6 | 0.064 (0.053, 0.077) |
| N-MeFOSE | 289 | 67.8 | 0.003 (0.003, 0.004) |
| PFHpS | 289 | 66.1 | 0.007 (0.006, 0.010) |
| PFUDA | 289 | 60.6 | 0.055 (0.045, 0.066) |
| PFMBA | 289 | 56.4 | 0.001 (0.001, 0.001) |
| 4:2 FTS | 289 | 54.0 | 0.002 (0.002, 0.003) |
| N-MeFOSAA | 289 | 51.2 | 0.016 (0.004, 0.008) |

LOD – limit of detection; Σ17PFAS – concentration sum of 17 PFAS with >50% detection

^1^ LODs are provided in supplemental table 2

Supplemental Table 6 – Concentrations of PFOA and PFOS (µg/L) according to sociodemographic characteristics

|  | **PFOA** | | | | **PFOS** | | | |
| --- | --- | --- | --- | --- | --- | --- | --- | --- |
|  | **n** | **GM (95% CI)** | **Overall**  **p-value** | **Pairwise**  **p-value^1^** | **n** | **GM (95% CI)** | **Overall**  **p-value** | **Pairwise**  **p-value^1^** |
| **Age (years)** | | | | | | | | |
| 32 – 39 | 82 | 0.448 (0.344, 0.584) | 0.67 |  | 82 | 1.621 (1.435, 1.831) | 0.14 |  |
| 40 – 44 | 111 | 0.545 (0.437, 0.678) |  |  | 111 | 1.821 (1.622, 2.045) |  |  |
| 45 – 49 | 71 | 0.485 (0.353, 0.667) |  |  | 71 | 1.484 (1.309, 1.683) |  |  |
| 50 and over | 25 | 0.577 (0.337, 0.989) |  |  | 25 | 1.741 (1.263, 2.400) |  |  |
| **Race and ethnicity^2^** | | | | | | | | |
| White | 258 | 0.538 (0.468, 0.619) | **0.03** |  | 258 | 1.697 (1.577, 1.825) | 0.24 |  |
| Other | 26 | 0.322 (0.160, 0.650) |  |  | 26 | 1.465 (1.125, 1.909) |  |  |
| **Country of Birth** | | | | | | | | |
| Canada | 248 | 0.510 (0.438, 0.594) | 0.82 |  | 248 | 1.660 (1.540, 1.789) | 0.52 |  |
| Other | 36 | 0.536 (0.356, 0.807) |  |  | 36 | 1.777 (1.449, 2.180) |  |  |
| **Body mass index (kg/m^2^)** | | | | | | | | |
| < 25 | 122 | 0.558 (0.455, 0.683) | 0.48 |  | 122 | 1.750 (1.577, 1.943) | 0.14 |  |
| 25 – 29 | 78 | 0.480 (0.364, 0.634) |  |  | 78 | 1.507 (1.336, 1.701) |  |  |
| ≥ 30 | 57 | 0.450 (0.312, 0.648) |  |  | 57 | 1.809 (1.504, 2.176) |  |  |
| **Smoking status** | | | | | | | | |
| Never | 192 | 0.541 (0.464, 0.631) | 0.35 |  | 192 | 1.649 (1.513, 1.797) | 0.35 |  |
| Former smoker | 75 | 0.448 (0.322, 0.624) |  |  | 75 | 1.635 (1.424, 1.877) |  |  |
| Current smoker | 22 | 0.399 (0.193, 0.826) |  |  | 22 | 1.993 (1.586, 2.505) |  |  |
| **Menopausal status** | | | | | | | | |
| Pre-menopause | 172 | 0.477 (0.399, 0.570) | 0.22 |  | 172 | 1.629 (1.502, 1.767) | 0.18 |  |
| Peri- or Post-menopausal | 54 | 0.458 (0.305, 0.689) |  |  | 54 | 1.590 (1.295, 1.954) |  |  |
| Using contraceptives | 46 | 0.666 (0.486, 0.911) |  |  | 46 | 1.941 (1.608, 2.343) |  |  |
| **Parity** | | | | | | | | |
| 1 | 32 | 0.856 (0.739, 0.992) | **0.02** | - | 32 | 1.754 (1.511, 2.036) | **0.046^3^** | - |
| 2 | 148 | 0.504 (0.413, 0.616) |  | 0.08 | 148 | 1.753 (1.587, 1.937) |  | 0.99 |
| ≥ 3 | 93 | 0.413 (0.311, 0.549) |  | **0.01** | 93 | 1.452 (1.282, 1.645) |  | 0.36 |
| **Time since last pregnancy (years)** | | | | | | | | |
| ≤ 4 | 33 | 0.261 (0.174, 0.392) | **<0.0001^4^** | - | 33 | 1.323 (1.080, 1.619) | **0.03** | - |
| > 4 – 8 | 98 | 0.385 (0.304, 0.487) |  | 0.72 | 98 | 1.585 (1.409, 1.782) |  | 0.35 |
| > 8 | 142 | 0.701 (0.576, 0.852) |  | **0.0001** | 142 | 1.774 (1.610, 1.956) |  | **0.03** |
| **Number of children breastfed** | | | | | | | | |
| ≤ 1 | 41 | 0.805 (0.623, 1.041) | **0.01** | - | 41 | 1.905 (1.608, 2.258) | **0.01** |  |
| 2 | 123 | 0.493 (0.393, 0.617) |  | 0.09 | 123 | 1.691 (1.518, 1.884) |  | 0.79 |
| ≥ 3 | 86 | 0.376 (0.280, 0.505) |  | **0.004** | 86 | 1.390 (1.225, 1.577) |  | **0.02** |
| **Lifetime duration of breastfeeding (years)** | | | | | | | | |
| ≤ 2 | 121 | 0.595 (0.476, 0.743) | **0.007** | **-** | 121 | 1.864 (1.681, 2.067) | **<0.0001** | **-** |
| > 2 – 4 | 90 | 0.466 (0.360, 0.604) |  | 0.48 | 90 | 1.509 (1.338, 1.701) |  | **0.03** |
| > 4 | 39 | 0.288 (0.194, 0.426) |  | **0.005** | 39 | 1.197 (0.997, 1.436) |  | **0.0001** |
| **Education** | | | | | | | | |
| College diploma or less | 70 | 0.501 (0.374, 0.672) | 0.97 |  | 70 | 1.491 (1.299, 1.712) | 0.07 |  |
| University degree | 219 | 0.504 (0.428, 0.594) |  |  | 219 | 1.730 (1.597, 1.874) |  |  |
| **Household Income ($CAD)** | | | | | | | | |
| ≤ 60,000 | 23 | 0.549 (0.295, 1.022) | 0.63 |  | 23 | 1.822 (1.344, 2.471) | 0.68 |  |
| 60 001 – 100 000 | 56 | 0.435 (0.317, 0.597) |  |  | 56 | 1.597 (1.371, 1.861) |  |  |
| > 100 000 | 204 | 0.514 (0.433, 0.609) |  |  | 204 | 1.668 (1.536, 1.813) |  |  |

^1^ Pairwise p-values are comparisons between categories and referent value and are corrected for multiple comparisons using the Bonferroni method. Pairwise comparisons were only calculated when the overall p-value was <0.05 and when there were multiple categories.

^2^ White: individuals who self-reported their race or ethnicity as White exclusively. Other: individuals who reported their race or ethnicity as anything other than White, including individuals who self-identified with more than one race or ethnicity

^3^ pairwise p = 0.050 between 2 and ≥ 3

^4^ pairwise p = 0.0004 between 4 – 8 and > 8

Supplemental Table 7 – Concentrations of PFHxS and PFNA (µg/L) according to sociodemographic characteristics

|  | **PFHxS** | | | | **PFNA** | | | |
| --- | --- | --- | --- | --- | --- | --- | --- | --- |
|  | **n** | **GM (95% CI)** | **Overall**  **p-value** | **Pairwise**  **p-value^1^** | **n** | **GM (95% CI)** | **Overall**  **p-value** | **Pairwise**  **p-value^1^** |
| **Age (years)** | | | | | | | | |
| 32 – 39 | 82 | 0.329 (0.283, 0.383) | 0.57 |  | 82 | 0.347 (0.306, 0.393) | 0.54 |  |
| 40 – 44 | 111 | 0.328 (0.282, 0.381) |  |  | 111 | 0.352 (0.309, 0.401) |  |  |
| 45 – 49 | 71 | 0.285 (0.245, 0.332) |  |  | 71 | 0.391 (0.345, 0.442) |  |  |
| 50 and over | 25 | 0.307 (0.233, 0.404) |  |  | 25 | 0.391 (0.313, 0.490) |  |  |
| **Race and ethnicity^2^** | | | | | | | | |
| White | 258 | 0.322 (0.295, 0.352) | 0.22 |  | 258 | 0.360 (0.333, 0.389) | 0.41 |  |
| Other | 26 | 0.269 (0.209, 0.347) |  |  | 26 | 0.399 (0.333, 0.479) |  |  |
| **Country of Birth** | | | | | | | | |
| Canada | 248 | 0.320 (0.292, 0.351) | 0.54 |  | 248 | 0.355 (0.328, 0.384) | 0.09 |  |
| Other | 36 | 0.296 (0.243, 0.361) |  |  | 36 | 0.427 (0.359, 0.507) |  |  |
| **Body mass index (kg/m^2^)** | | | | | | | | |
| < 25 | 122 | 0.321 (0.285, 0.362) | 0.18 |  | 122 | 0.384 (0.338, 0.436) | 0.50 |  |
| 25 – 29 | 78 | 0.291 (0.243, 0.347) |  |  | 78 | 0.354 (0.314, 0.399) |  |  |
| ≥ 30 | 57 | 0.369 (0.300, 0.454) |  |  | 57 | 0.345 (0.297, 0.400) |  |  |
| **Smoking status** | | | | | | | | |
| Never | 192 | 0.306 (0.276, 0.339) | 0.38 |  | 192 | 0.361 (0.328, 0.396) | 0.84 |  |
| Former smoker | 75 | 0.321 (0.270, 0.382) |  |  | 75 | 0.361 (0.320, 0.406) |  |  |
| Current smoker | 22 | 0.382 (0.300, 0.486) |  |  | 22 | 0.390 (0.324, 0.470) |  |  |
| **Menopausal status** | | | | | | | | |
| Pre-menopause | 172 | 0.304 (0.273, 0.338) | 0.32 |  | 172 | 0.347 (0.316, 0.382) | 0.06 |  |
| Peri- or Post-menopausal | 54 | 0.298 (0.242, 0.366) |  |  | 54 | 0.350 (0.293, 0.417) |  |  |
| Using contraceptives | 46 | 0.361 (0.290, 0.448) |  |  | 46 | 0.442 (0.377, 0.518) |  |  |
| **Parity** | | | | | | | | |
| 1 | 32 | 0.456 (0.365, 0.571) | **0.001** | **-** | 32 | 0.331 (0.228, 0.480) | 0.25 |  |
| 2 | 148 | 0.321 (0.285, 0.363) |  | **0.04** | 148 | 0.383 (0.353, 0.415) |  |  |
| ≥ 3 | 93 | 0.266 (0.231, 0.306) |  | **0.001** | 93 | 0.340 (0.300, 0.386) |  |  |
| **Time since last pregnancy (years)** | | | | | | | | |
| ≤ 4 | 33 | 0.230 (0.180, 0.293) | **0.003** | - | 33 | 0.285 (0.231, 0.352) | 0.06 |  |
| > 4 – 8 | 98 | 0.291 (0.252, 0.335) |  | 0.46 | 98 | 0.370 (0.327, 0.418) |  |  |
| > 8 | 142 | 0.356 (0.316, 0.400) |  | **0.005** | 142 | 0.376 (0.340, 0.417) |  |  |
| **Number of children breastfed** | | | | | | | | |
| ≤ 1 | 41 | 0.444 (0.359, 0.550) | **0.0003** | **-** | 41 | 0.359 (0.266, 0.483) | 0.13 |  |
| 2 | 123 | 0.317 (0.279, 0.361) |  | **0.03** | 123 | 0.381 (0.348, 0.417) |  |  |
| ≥ 3 | 86 | 0.255 (0.220, 0.297) |  | **0.0002** | 86 | 0.320 (0.283, 0.361) |  |  |
| **Lifetime duration of breastfeeding (years)** | | | | | | | | |
| ≤ 2 | 121 | 0.371 (0.327, 0.421) | **<0.0001** | **-** | 121 | 0.371 (0.333, 0.415) | 0.09 |  |
| > 2 – 4 | 90 | 0.290 (0.250, 0.335) |  | **0.04** | 90 | 0.365 (0.321, 0.415) |  |  |
| > 4 | 39 | 0.213 (0.170, 0.266) |  | **<0.0001** | 39 | 0.292 (0.240, 0.354) |  |  |
| **Education** | | | | | | | | |
| College diploma or less | 70 | 0.320 (0.273, 0.375) | 0.85 |  | 70 | 0.331 (0.292, 0.376) | 0.15 |  |
| University degree | 219 | 0.314 (0.284, 0.346) |  |  | 219 | 0.374 (0.344, 0.406) |  |  |
| **Household Income ($CAD)** | | | | | | | | |
| ≤ 60,000 | 23 | 0.439 (0.319, 0.605) | **0.03** | **-** | 23 | 0.398 (0.325, 0.488) | 0.70 |  |
| 60 001 – 100 000 | 56 | 0.271 (0.223, 0.330) |  | **0.02** | 56 | 0.351 (0.302, 0.406) |  |  |
| > 100 000 | 204 | 0.315 (0.286, 0.348) |  | 0.11 | 204 | 0.362 (0.331, 0.395) |  |  |

^1^ Pairwise p-values are comparisons between categories and referent value and are corrected for multiple comparisons using the Bonferroni method. Pairwise comparisons were only calculated when the overall p-value was <0.05 and when there were multiple categories.

^2^ White: individuals who self-reported their race or ethnicity as White exclusively. Other: individuals who reported their race or ethnicity as anything other than White, including individuals who self-identified with more than one race or ethnicity

Supplemental Table 8 – Concentrations of PFHpS and PFUDA (µg/L) according to sociodemographic characteristics

|  | **PFHpS** | | | | **PFUDA** | | | |
| --- | --- | --- | --- | --- | --- | --- | --- | --- |
|  | **n** | **GM (95% CI)** | **Overall**  **p-value** | **Pairwise**  **p-value^1^** | **n** | **GM (95% CI)** | **Overall**  **p-value** | **Pairwise**  **p-value^1^** |
| **Age (years)** | | | | | | | | |
| 32 – 39 | 82 | 0.011 (0.008, 0.016) | 0.29 |  | 82 | 0.085 (0.069, 0.105) | 0.33 |  |
| 40 – 44 | 111 | 0.013 (0.009, 0.017) |  |  | 111 | 0.079 (0.064, 0.097) |  |  |
| 45 – 49 | 71 | 0.009 (0.007, 0.013) |  |  | 71 | 0.091 (0.070, 0.118) |  |  |
| 50 and over | 25 | 0.017 (0.010, 0.029) |  |  | 25 | 0.119 (0.089, 0.159) |  |  |
| **Race and ethnicity^2^** | | | | | | | | |
| White | 258 | 0.012 (0.010, 0.014) | 0.55 |  | 258 | 0.083 (0.073, 0.095) | 0.08 |  |
| Other | 26 | 0.010 (0.006, 0.018) |  |  | 26 | 0.122 (0.094, 0.160) |  |  |
| **Country of Birth** | | | | | | | | |
| Canada | 248 | 0.012 (0.010, 0.015) | 0.43 |  | 248 | 0.084 (0.074, 0.096) | 0.33 |  |
| Other | 36 | 0.010 (0.006, 0.016) |  |  | 36 | 0.101 (0.070, 0.147) |  |  |
| **Body mass index (kg/m^2^)** | | | | | | | | |
| < 25 | 122 | 0.011 (0.008, 0.014) | 0.30 |  | 122 | 0.105 (0.087, 0.126) | **0.006** | - |
| 25 – 29 | 78 | 0.012 (0.009, 0.016) |  |  | 78 | 0.065 (0.050, 0.086) |  | **0.007** |
| ≥ 30 | 57 | 0.016 (0.010, 0.024) |  |  | 57 | 0.074 (0.059, 0.094) |  | 0.13 |
| **Smoking status** | | | | | | | | |
| Never | 192 | 0.012 (0.010, 0.015) | 0.30 |  | 192 | 0.088 (0.075, 0.103) | 0.63 |  |
| Former smoker | 75 | 0.010 (0.007, 0.014) |  |  | 75 | 0.089 (0.075, 0.106) |  |  |
| Current smoker | 22 | 0.017 (0.009, 0.033) |  |  | 22 | 0.070 (0.041, 0.119) |  |  |
| **Menopausal status** | | | | | | | | |
| Pre-menopause | 172 | 0.011 (0.009, 0.013) | 0.47 |  | 172 | 0.086 (0.075, 0.099) | 0.41 |  |
| Peri- or Post-menopausal | 54 | 0.011 (0.007, 0.017) |  |  | 54 | 0.073 (0.049, 0.107) |  |  |
| Using contraceptives | 46 | 0.015 (0.009, 0.024) |  |  | 46 | 0.096 (0.070, 0.131) |  |  |
| **Parity** | | | | | | | | |
| 1 | 32 | 0.016 (0.009, 0.027) | **0.007^3^** | - | 32 | 0.086 (0.067, 0.110) | 0.99 |  |
| 2 | 148 | 0.014 (0.011, 0.018) |  | 0.99 | 148 | 0.085 (0.071, 0.101) |  |  |
| ≥ 3 | 93 | 0.008 (0.006, 0.011) |  | 0.07 | 93 | 0.086 (0.069, 0.107) |  |  |
| **Time since last pregnancy (years)** | | | | | | | | |
| ≤ 4 | 33 | 0.004 (0.002, 0.006) | **<0.0001^4^** | - | 33 | 0.094 (0.065, 0.135) | 0.41 |  |
| > 4 – 8 | 98 | 0.010 (0.007, 0.013) |  | **0.006** | 98 | 0.076 (0.062, 0.094) |  |  |
| > 8 | 142 | 0.017 (0.014, 0.022) |  | **<0.0001** | 142 | 0.090 (0.076, 0.107) |  |  |
| **Number of children breastfed** | | | | | | | | |
| ≤ 1 | 41 | 0.022 (0.014, 0.034) | **0.005** | - | 41 | 0.087 (0.070, 0.107) | 0.86 |  |
| 2 | 123 | 0.012 (0.010, 0.016) |  | 0.09 | 123 | 0.081 (0.065, 0.100) |  |  |
| ≥ 3 | 86 | 0.009 (0.006, 0.012) |  | **0.003** | 86 | 0.077 (0.062, 0.097) |  |  |
| **Lifetime duration of breastfeeding (years)** | | | | | | | | |
| ≤ 2 | 121 | 0.014 (0.011, 0.019) | **0.02** | - | 121 | 0.075 (0.062, 0.091) | 0.50 |  |
| > 2 – 4 | 90 | 0.012 (0.009, 0.017) |  | 0.99 | 90 | 0.089 (0.072, 0.112) |  |  |
| > 4 | 39 | 0.007 (0.004, 0.011) |  | **0.01** | 39 | 0.078 (0.055, 0.109) |  |  |
| **Education** | | | | | | | | |
| College diploma or less | 70 | 0.013 (0.009, 0.018) | 0.42 |  | 70 | 0.073 (0.058, 0.093) | 0.12 |  |
| University degree | 219 | 0.011 (0.009, 0.014) |  |  | 219 | 0.091 (0.079, 0.105) |  |  |
| **Household Income ($CAD)** | | | | | | | | |
| ≤ 60,000 | 23 | 0.015 (0.007, 0.030) | 0.67 |  | 23 | 0.076 (0.047, 0.124) | 0.71 |  |
| 60 001 – 100 000 | 56 | 0.012 (0.008, 0.017) |  |  | 56 | 0.082 (0.064, 0.105) |  |  |
| > 100 000 | 204 | 0.011 (0.009, 0.014) |  |  | 204 | 0.089 (0.077, 0.104) |  |  |

^1^ Pairwise p-values are comparisons between categories and referent value and are corrected for multiple comparisons using the Bonferroni method. Pairwise comparisons were only calculated when the overall p-value was <0.05 and when there were multiple categories.

^2^ White: individuals who self-reported their race or ethnicity as White exclusively. Other: individuals who reported their race or ethnicity as anything other than White, including individuals who self-identified with more than one race or ethnicity

^3^ pairwise p = 0.01 for 2 vs ≥ 3

^4^ pairwise p = 0.004 for 4 – 8 years vs. > 8 years

Supplemental Table 9 – Concentrations of PFDA and PFBA (µg/L) according to sociodemographic characteristics

|  | **PFDA** | | | | **PFBA** | | | |
| --- | --- | --- | --- | --- | --- | --- | --- | --- |
|  | **n** | **GM (95% CI)** | **Overall**  **p-value** | **Pairwise**  **p-value^1^** | **n** | **GM (95% CI)** | **Overall**  **p-value** | **Pairwise**  **p-value^1^** |
| **Age (years)** | | | | | | | | |
| 32 – 39 | 82 | 0.135 (0.116, 0.157) | 0.39 |  | 82 | 0.092 (0.073, 0.115) | 0.66 |  |
| 40 – 44 | 111 | 0.133 (0.117, 0.151) |  |  | 111 | 0.086 (0.068, 0.109) |  |  |
| 45 – 49 | 71 | 0.153 (0.130, 0.180) |  |  | 71 | 0.087 (0.061, 0.122) |  |  |
| 50 and over | 25 | 0.158 (0.126, 0.199) |  |  | 25 | 0.119 (0.082, 0.174) |  |  |
| **Race and ethnicity^2^** | | | | | | | | |
| White | 258 | 0.141 (0.130, 0.153) | 0.97 |  | 258 | 0.091 (0.079, 0.105) | 0.53 |  |
| Other | 26 | 0.140 (0.111, 0.177) |  |  | 26 | 0.078 (0.041, 0.148) |  |  |
| **Country of Birth** | | | | | | | | |
| Canada | 248 | 0.136 (0.125, 0.149) | **0.03** |  | 248 | 0.090 (0.078, 0.104) | 0.98 |  |
| Other | 36 | 0.177 (0.147, 0.213) |  |  | 36 | 0.089 (0.051, 0.155) |  |  |
| **Body mass index (kg/m^2^)** | | | | | | | | |
| < 25 | 122 | 0.160 (0.142, 0.180) | **0.009** | - | 122 | 0.100 (0.082, 0.122) | 0.11 |  |
| 25 – 29 | 78 | 0.121 (0.103, 0.141) |  | **0.01** | 78 | 0.081 (0.060, 0.109) |  |  |
| ≥ 30 | 57 | 0.129 (0.110, 0.153) |  | 0.14 | 57 | 0.067 (0.048, 0.094) |  |  |
| **Smoking status** | | | | | | | | |
| Never | 192 | 0.137 (0.125, 0.151) | 0.73 |  | 192 | 0.094 (0.080, 0.112) | **0.03^3^** |  |
| Former smoker | 75 | 0.145 (0.123, 0.170) |  |  | 75 | 0.098 (0.076, 0.127) |  | 0.99 |
| Current smoker | 22 | 0.151 (0.114, 0.199) |  |  | 22 | 0.047 (0.024, 0.093) |  | **0.03** |
| **Menopausal status** | | | | | | | | |
| Pre-menopause | 172 | 0.135 (0.123, 0.149) | 0.12 |  | 172 | 0.083 (0.068, 0.100) | 0.46 |  |
| Peri- or Post-menopausal | 54 | 0.133 (0.110, 0.161) |  |  | 54 | 0.105 (0.076, 0.145) |  |  |
| Using contraceptives | 46 | 0.169 (0.134, 0.213) |  |  | 46 | 0.092 (0.068, 0.126) |  |  |
| **Parity** | | | | | | | | |
| 1 | 32 | 0.131 (0. 105, 0.164) | 0.75 |  | 32 | 0.111 (0.074, 0.168) | 0.39 |  |
| 2 | 148 | 0.140 (0.125, 0.155) |  |  | 148 | 0.082 (0.067, 0.100) |  |  |
| ≥ 3 | 93 | 0.145 (0.125, 0.168) |  |  | 93 | 0.092 (0.073, 0.116) |  |  |
| **Time since last pregnancy (years)** | | | | | | | | |
| ≤ 4 | 33 | 0.136 (0.108, 0.171) | 0.80 |  | 33 | 0.131 (0.087, 0.197) | 0.06 |  |
| > 4 – 8 | 98 | 0.146 (0.127, 0.166) |  |  | 98 | 0.074 (0.054, 0.094) |  |  |
| > 8 | 142 | 0.138 (0.124, 0.154) |  |  | 142 | 0.091 (0.075, 0.111) |  |  |
| **Number of children breastfed** | | | | | | | | |
| ≤ 1 | 41 | 0.133 (0.108, 0.164) | 0.76 |  | 41 | 0.091 (0.060, 0.137) | 0.84 |  |
| 2 | 123 | 0.140 (0.125, 0.158) |  |  | 123 | 0.096 (0.077, 0.119) |  |  |
| ≥ 3 | 86 | 0.132 (0.114, 0.151) |  |  | 86 | 0.086 (0.067, 0.111) |  |  |
| **Lifetime duration of breastfeeding (years)** | | | | | | | | |
| ≤ 2 | 121 | 0.131 (0.117, 0.148) | 0.69 |  | 121 | 0.093 (0.075, 0.116) | 0.97 |  |
| > 2 – 4 | 90 | 0.142 (0.124, 0.163) |  |  | 90 | 0.089 (0.070, 0.115) |  |  |
| > 4 | 39 | 0.138 (0.112, 0.170) |  |  | 39 | 0.091 (0.062, 0.134) |  |  |
| **Education** | | | | | | | | |
| College diploma or less | 70 | 0.124 (0.105, 0.147) | 0.08 |  | 70 | 0.073 (0.055, 0.098) | 0.10 |  |
| University degree | 219 | 0.146 (0.133, 0.159) |  |  | 219 | 0.096 (0.082, 0.113) |  |  |
| **Household Income ($CAD)** | | | | | | | | |
| ≤ 60,000 | 23 | 0.127 (0.090, 0.179) | 0.48 |  | 23 | 0.057 (0.028, 0.119) | 0.17 |  |
| 60 001 – 100 000 | 56 | 0.131 (0.110, 0.155) |  |  | 56 | 0.099 (0.075, 0.131) |  |  |
| > 100 000 | 204 | 0.144 (0.132, 0.158) |  |  | 204 | 0.092 (0.078, 0.108) |  |  |

^1^ Pairwise p-values are comparisons between categories and referent value and are corrected for multiple comparisons using the Bonferroni method. Pairwise comparisons were only calculated when the overall p-value was <0.05 and when there were multiple categories.

^2^ White: individuals who self-reported their race or ethnicity as White exclusively. Other: individuals who reported their race or ethnicity as anything other than White, including individuals who self-identified with more than one race or ethnicity

^3^ pairwise p = 0.03 for former smoker vs. current smoker

Supplemental Table 10 – Concentrations of PFOSA and PFPeA (µg/L) according to sociodemographic characteristics

|  | **PFOSA** | | | | **PFPeA** | | | |
| --- | --- | --- | --- | --- | --- | --- | --- | --- |
|  | **n** | **GM (95% CI)** | **Overall**  **p-value** | **Pairwise**  **p-value^1^** | **n** | **GM (95% CI)** | **Overall**  **p-value** | **Pairwise**  **p-value^1^** |
| **Age (years)** | | | | | | | | |
| 32 – 39 | 82 | 0.002 (0.001, 0.002) | 0.09 |  | 82 | 0.008 (0.005, 0.012) | 0.03^3^ | - |
| 40 – 44 | 111 | 0.002 (0.002, 0.002) |  |  | 111 | 0.013 (0.009, 0.017) |  | 0.45 |
| 45 – 49 | 71 | 0.001 (0.001, 0.002) |  |  | 71 | 0.006 (0.004, 0.009) |  | 0.99 |
| 50 and over | 25 | 0.002 (0.001, 0.003) |  |  | 25 | 0.007 (0.003, 0.004) |  | 0.99 |
| **Race and ethnicity^2^** | | | | | | | | |
| White | 258 | 0.002 (0.002, 0.002) | 0.85 |  | 258 | 0.009 (0.007, 0.011) | 0.67 |  |
| Other | 26 | 0.002 (0.001, 0.002) |  |  | 26 | 0.008 (0.004, 0.016) |  |  |
| **Country of Birth** | | | | | | | | |
| Canada | 248 | 0.002 (0.002, 0.002) | 0.70 |  | 248 | 0.009 (0.007, 0.011) | 0.99 |  |
| Other | 36 | 0.002 (0.001, 0.002) |  |  | 36 | 0.009 (0.005, 0.016) |  |  |
| **Body mass index (kg/m^2^)** | | | | | | | | |
| < 25 | 122 | 0.002 (0.002, 0.002) | 0.70 |  | 122 | 0.010 (0.007, 0.013) | 0.83 |  |
| 25 – 29 | 78 | 0.002 (0.001, 0.002) |  |  | 78 | 0.010 (0.007, 0.015) |  |  |
| ≥ 30 | 57 | 0.002 (0.001, 0.002) |  |  | 57 | 0.009 (0.005, 0.014) |  |  |
| **Smoking status** | | | | | | | | |
| Never | 192 | 0.002 (0.001, 0.002) | 0.27 |  | 192 | 0.010 (0.008, 0.012) | 0.36 |  |
| Former smoker | 75 | 0.002 (0.002, 0.002) |  |  | 75 | 0.007 (0.005, 0.011) |  |  |
| Current smoker | 22 | 0.002 (0.001, 0.003) |  |  | 22 | 0.007 (0.003, 0.015) |  |  |
| **Menopausal status** | | | | | | | | |
| Pre-menopause | 172 | 0.002 (0.002, 0.002) | 0.97 |  | 172 | 0.010 (0.008, 0.013) | 0.41 |  |
| Peri- or Post-menopausal | 54 | 0.002 (0.001, 0.002) |  |  | 54 | 0.008 (0.005, 0.013) |  |  |
| Using contraceptives | 46 | 0.002 (0.001, 0.002) |  |  | 46 | 0.007 (0.004, 0.011) |  |  |
| **Parity** | | | | | | | | |
| 1 | 32 | 0.001 (0.001, 0.002) | 0.17 |  | 32 | 0.010 (0.006, 0.019) | 0.11 |  |
| 2 | 148 | 0.002 (0.001, 0.002) |  |  | 148 | 0.008 (0.006, 0.010) |  |  |
| ≥ 3 | 93 | 0.002 (0.002, 0.002) |  |  | 93 | 0.012 (0.008, 0.017) |  |  |
| **Time since last pregnancy (years)** | | | | | | | | |
| ≤ 4 | 33 | 0.002 (0.001, 0.002) | 0.97 |  | 33 | 0.011 (0.006, 0.019) | 0.86 |  |
| > 4 – 8 | 98 | 0.002 (0.001, 0.002) |  |  | 98 | 0.009 (0.006, 0.013) |  |  |
| > 8 | 142 | 0.002 (0.002, 0.002) |  |  | 142 | 0.009 (0.007, 0.012) |  |  |
| **Number of children breastfed** | | | | | | | | |
| ≤ 1 | 41 | 0.001 (0.001, 0.002) | 0.09 |  | 41 | 0.010 (0.006, 0.017) | 0.34 |  |
| 2 | 123 | 0.002 (0.001, 0.002) |  |  | 123 | 0.008 (0.006, 0.011) |  |  |
| ≥ 3 | 86 | 0.002 (0.002, 0.003) |  |  | 86 | 0.012 (0.008, 0.017) |  |  |
| **Lifetime duration of breastfeeding (years)** | | | | | | | | |
| ≤ 2 | 121 | 0.002 (0.001, 0.002) | 0.04 | - | 121 | 0.010 (0.007, 0.013) | 0.50 |  |
| > 2 – 4 | 90 | 0.002 (0.002, 0.002) |  | 0.23 | 90 | 0.008 (0.006, 0.012) |  |  |
| > 4 | 39 | 0.002 (0.002, 0.003) |  | 0.07 | 39 | 0.012 (0.007, 0.021) |  |  |
| **Education** | | | | | | | | |
| College diploma or less | 70 | 0.002 (0.001, 0.002) | 0.75 |  | 70 | 0.009 (0.006, 0.013) | 0.87 |  |
| University degree | 219 | 0.002 (0.002, 0.002) |  |  | 219 | 0.009 (0.007, 0.011) |  |  |
| **Household Income ($CAD)** | | | | | | | | |
| ≤ 60,000 | 23 | 0.002 (0.001, 0.003) | 0.98 |  | 23 | 0.011 (0.006, 0.022) | 0.72 |  |
| 60 001 – 100 000 | 56 | 0.002 (0.001, 0.002) |  |  | 56 | 0.008 (0.005, 0.012) |  |  |
| > 100 000 | 204 | 0.002 (0.002, 0.002) |  |  | 204 | 0.009 (0.007, 0.011) |  |  |

^1^ Pairwise p-values are comparisons between categories and referent value and are corrected for multiple comparisons using the Bonferroni method. Pairwise comparisons were only calculated when the overall p-value was <0.05 and when there were multiple categories.

^2^ White: individuals who self-reported their race or ethnicity as White exclusively. Other: individuals who reported their race or ethnicity as anything other than White, including individuals who self-identified with more than one race or ethnicity

^3^ pairwise p = 0.026 for 40 – 44 vs. 45 – 49

Supplemental Table 11 – Concentrations of PFMBA and MeFOSAA (µg/L) according to sociodemographic characteristics

|  | **PFMBA** | | | | **MeFOSAA** | | | |
| --- | --- | --- | --- | --- | --- | --- | --- | --- |
|  | **n** | **GM (95% CI)** | **Overall**  **p-value** | **Pairwise**  **p-value^1^** | **n** | **GM (95% CI)** | **Overall**  **p-value** | **Pairwise**  **p-value^1^** |
| **Age (years)** | | | | | | | | |
| 32 – 39 | 82 | 0.001 (0.001, 0.001) | 0.24 |  | 82 | 0.013 (0.010, 0.018) | 0.32 |  |
| 40 – 44 | 111 | 0.001 (0.001, 0.002) |  |  | 111 | 0.012 (0.009, 0.014) |  |  |
| 45 – 49 | 71 | 0.001 (0.001, 0.002) |  |  | 71 | 0.013 (0.009, 0.018) |  |  |
| 50 and over | 25 | 0.001 (0.001, 0.001) |  |  | 25 | 0.019 (0.011, 0.033) |  |  |
| **Race and ethnicity^2^** | | | | | | | | |
| White | 258 | 0.001 (0.001, 0.001) | 0.93 |  | 258 | 0.013 (0.011, 0.015) | 0.83 |  |
| Other | 26 | 0.001 (0.001, 0.002) |  |  | 26 | 0.014 (0.008, 0.023) |  |  |
| **Country of Birth** | | | | | | | | |
| Canada | 248 | 0.001 (0.001, 0.001) | 0.87 |  | 248 | 0.013 (0.011, 0.015) | 0.81 |  |
| Other | 36 | 0.001 (0.001, 0.002) |  |  | 36 | 0.014 (0.009, 0.021) |  |  |
| **Body mass index (kg/m^2^)** | | | | | | | | |
| < 25 | 122 | 0.001 (0.001, 0.002) | 0.64 |  | 122 | 0.013 (0.010, 0.016) | 0.89 |  |
| 25 – 29 | 78 | 0.001 (0.001, 0.001) |  |  | 78 | 0.013 (0.010, 0.018) |  |  |
| ≥ 30 | 57 | 0.001 (0.001, 0.002) |  |  | 57 | 0.012 (0.009, 0.017) |  |  |
| **Smoking status** | | | | | | | | |
| Never | 192 | 0.001 (0.001, 0.001) | 0.50 |  | 192 | 0.013 (0.011, 0.016) | 0.30 |  |
| Former smoker | 75 | 0.001 (0.001, 0.002) |  |  | 75 | 0.011 (0.009, 0.015) |  |  |
| Current smoker | 22 | 0.001 (0.001, 0.002) |  |  | 22 | 0.018 (0.010, 0.031) |  |  |
| **Menopausal status** | | | | | | | | |
| Pre-menopause | 172 | 0.001 (0.001, 0.001) | 0.62 |  | 172 | 0.012 (0.010, 0.014) | 0.11 |  |
| Peri- or Post-menopausal | 54 | 0.001 (0.001, 0.001) |  |  | 54 | 0.013 (0.009, 0.017) |  |  |
| Using contraceptives | 46 | 0.001 (0.001, 0.001) |  |  | 46 | 0.018 (0.012, 0.026) |  |  |
| **Parity** | | | | | | | | |
| 1 | 32 | 0.001 (0.001, 0.002) | 0.33 |  | 32 | 0.012 (0.008, 0.018) | 0.76 |  |
| 2 | 148 | 0.001 (0.001, 0.001) |  |  | 148 | 0.014 (0.011, 0.017) |  |  |
| ≥ 3 | 93 | 0.001 (0.001, 0.002) |  |  | 93 | 0.013 (0.010, 0.016) |  |  |
| **Time since last pregnancy (years)** | | | | | | | | |
| ≤ 4 | 33 | 0.001 (0.001, 0.001) | 0.41 |  | 33 | 0.015 (0.010, 0.023) | 0.72 |  |
| > 4 – 8 | 98 | 0.001 (0.001, 0.001) |  |  | 98 | 0.013 (0.010, 0.016) |  |  |
| > 8 | 142 | 0.001 (0.001, 0.001) |  |  | 142 | 0.013 (0.011, 0.016) |  |  |
| **Number of children breastfed** | | | | | | | | |
| ≤ 1 | 41 | 0.001 (0.001, 0.002) | 0.40 |  | 41 | 0.014 (0.010, 0.021) | 0.95 |  |
| 2 | 123 | 0.001 (0.001, 0.001) |  |  | 123 | 0.013 (0.011, 0.017) |  |  |
| ≥ 3 | 86 | 0.001 (0.001, 0.002) |  |  | 86 | 0.013 (0.010, 0.017) |  |  |
| **Lifetime duration of breastfeeding (years)** | | | | | | | | |
| ≤ 2 | 121 | 0.001 (0.001, 0.001) | **0.004** | **-** | 121 | 0.012 (0.009, 0.014) | 0.17 |  |
| > 2 – 4 | 90 | 0.001 (0.001, 0.002) |  | 0.41 | 90 | 0.016 (0.012, 0.020) |  |  |
| > 4 | 39 | 0.002 (0.001, 0.002) |  | **0.003** | 39 | 0.015 (0.010, 0.021) |  |  |
| **Education** | | | | | | | | |
| College diploma or less | 70 | 0.001 (0.001, 0.002) | 0.29 |  | 70 | 0.014 (0.010, 0.018) | 0.67 |  |
| University degree | 219 | 0.001 (0.001, 0.001) |  |  | 219 | 0.013 (0.011, 0.015) |  |  |
| **Household Income ($CAD)** | | | | | | | | |
| ≤ 60,000 | 23 | 0.002 (0.001, 0.002) | 0.38 |  | 23 | 0.012 (0.007, 0.021) | 0.16 |  |
| 60 001 – 100 000 | 56 | 0.001 (0.001, 0.002) |  |  | 56 | 0.017 (0.012, 0.025) |  |  |
| > 100 000 | 204 | 0.001 (0.001, 0.001) |  |  | 204 | 0.012 (0.010, 0.014) |  |  |

^1^ Pairwise p-values are comparisons between categories and referent value and are corrected for multiple comparisons using the Bonferroni method. Pairwise comparisons were only calculated when the overall p-value was <0.05 and when there were multiple categories.

^2^ White: individuals who self-reported their race or ethnicity as White exclusively. Other: individuals who reported their race or ethnicity as anything other than White, including individuals who self-identified with more than one race or ethnicity

Supplemental Table 12 – Concentrations of N-MeFOSE and N-EtFOSE (µg/L) according to sociodemographic characteristics

|  | **N-MeFOSE** | | | | **N-EtFOSE** | | | |
| --- | --- | --- | --- | --- | --- | --- | --- | --- |
|  | **n** | **GM (95% CI)** | **Overall**  **p-value** | **Pairwise**  **p-value^1^** | **n** | **GM (95% CI)** | **Overall**  **p-value** | **Pairwise**  **p-value^1^** |
| **Age (years)** | | | | | | | | |
| 32 – 39 | 82 | 0.005 (0.004, 0.007) | 0.56 |  | 82 | 0.002 (0.002, 0.003) | 0.32 |  |
| 40 – 44 | 111 | 0.006 (0.005, 0.008) |  |  | 111 | 0.002 (0.002, 0.003) |  |  |
| 45 – 49 | 71 | 0.006 (0.004, 0.008) |  |  | 71 | 0.003 (0.002, 0.004) |  |  |
| 50 and over | 25 | 0.007 (0.004, 0.012) |  |  | 25 | 0.004 (0.002, 0.008) |  |  |
| **Race and ethnicity^2^** | | | | | | | | |
| White | 258 | 0.005 (0.004, 0.006) | **0.04** |  | 258 | 0.003 (0.002, 0.003) | **0.04** |  |
| Other | 26 | 0.010 (0.006, 0.018) |  |  | 26 | 0.005 (0.002, 0.008) |  |  |
| **Country of Birth** | | | | | | | | |
| Canada | 248 | 0.005 (0.005, 0.007) | 0.80 |  | 248 | 0.003 (0.002, 0.003) | 0.89 |  |
| Other | 36 | 0.006 (0.003, 0.010) |  |  | 36 | 0.003 (0.002, 0.004) |  |  |
| **Body mass index (kg/m^2^)** | | | | | | | | |
| < 25 | 122 | 0.006 (0.005, 0.007) | 0.48 |  | 122 | 0.003 (0.002, 0.003) | 0.99 |  |
| 25 – 29 | 78 | 0.006 (0.004, 0.008) |  |  | 78 | 0.003 (0.002, 0.004) |  |  |
| ≥ 30 | 57 | 0.004 (0.003, 0.006) |  |  | 57 | 0.003 (0.002, 0.004) |  |  |
| **Smoking status** | | | | | | | | |
| Never | 192 | 0.005 (0.005, 0.007) | 0.99 |  | 192 | 0.003 (0.002, 0.003) | 0.39 |  |
| Former smoker | 75 | 0.006 (0.004, 0.008) |  |  | 75 | 0.002 (0.002, 0.003) |  |  |
| Current smoker | 22 | 0.006 (0.004, 0.011) |  |  | 22 | 0.005 (0.005, 0.007) |  |  |
| **Menopausal status** | | | | | | | | |
| Pre-menopause | 172 | 0.006 (0.005, 0.007) | 0.82 |  | 172 | 0.002 (0.002, 0.003) | 0.41 |  |
| Peri- or Post-menopausal | 54 | 0.006 (0.004, 0.009) |  |  | 54 | 0.003 (0.002, 0.005) |  |  |
| Using contraceptives | 46 | 0.005 (0.003, 0.008) |  |  | 46 | 0.003 (0.002, 0.004) |  |  |
| **Parity** | | | | | | | | |
| 1 | 32 | 0.005 (0.003, 0.008) | 0.83 |  | 32 | 0.003 (0.002, 0.005) | 0.25 |  |
| 2 | 148 | 0.006 (0.004, 0.007) |  |  | 148 | 0.003 (0.002, 0.004) |  |  |
| ≥ 3 | 93 | 0.006 (0.004, 0.008) |  |  | 93 | 0.002 (0.002, 0.003) |  |  |
| **Time since last pregnancy (years)** | | | | | | | | |
| ≤ 4 | 33 | 0.007 (0.004, 0.011) | 0.55 |  | 33 | 0.002 (0.001, 0.003) | **0.02** | - |
| > 4 – 8 | 98 | 0.006 (0.004, 0.008) |  |  | 98 | 0.002 (0.002, 0.003) |  | 0.86 |
| > 8 | 142 | 0.005 (0.004, 0.006) |  |  | 142 | 0.003 (0.003, 0.004) |  | **0.04** |
| **Number of children breastfed** | | | | | | | | |
| ≤ 1 | 41 | 0.004 (0.003, 0.007) | 0.36 |  | 41 | 0.004 (0.002, 0.006) | 0.15 |  |
| 2 | 123 | 0.005 (0.004, 0.007) |  |  | 123 | 0.003 (0.002, 0.003) |  |  |
| ≥ 3 | 86 | 0.006 (0.004, 0.008) |  |  | 86 | 0.002 (0.002, 0.003) |  |  |
| **Lifetime duration of breastfeeding (years)** | | | | | | | | |
| ≤ 2 | 121 | 0.005 (0.004, 0.006) | 0.16 |  | 121 | 0.003 (0.002, 0.003) | 0.82 |  |
| > 2 – 4 | 90 | 0.006 (0.004, 0.008) |  |  | 90 | 0.003 (0.002, 0.003) |  |  |
| > 4 | 39 | 0.008 (0.005, 0.012) |  |  | 39 | 0.003 (0.002, 0.005) |  |  |
| **Education** | | | | | | | | |
| College diploma or less | 70 | 0.005 (0.003, 0.007) | 0.30 |  | 70 | 0.003 (0.002, 0.004) | 0.92 |  |
| University degree | 219 | 0.006 (0.005, 0.007) |  |  | 219 | 0.003 (0.002, 0.003) |  |  |
| **Household Income ($CAD)** | | | | | | | | |
| ≤ 60,000 | 23 | 0.006 (0.003, 0.013) | 0.99 |  | 23 | 0.003 (0.002, 0.006) | 0.62 |  |
| 60 001 – 100 000 | 56 | 0.006 (0.004, 0.008) |  |  | 56 | 0.003 (0.002, 0.004) |  |  |
| > 100 000 | 204 | 0.005 (0.004, 0.007) |  |  | 204 | 0.002 (0.002, 0.003) |  |  |

^1^ Pairwise p-values are comparisons between categories and referent value and are corrected for multiple comparisons using the Bonferroni method. Pairwise comparisons were only calculated when the overall p-value was <0.05 and when there were multiple categories.

^2^ White: individuals who self-reported their race or ethnicity as White exclusively. Other: individuals who reported their race or ethnicity as anything other than White, including individuals who self-identified with more than one race or ethnicity

Supplemental Table 13 – Concentrations of 7:3 FTCA and 4:2 FTS (µg/L) according to sociodemographic characteristics

|  | **7:3 FTCA** | | | | **4:2 FTS** | | | |
| --- | --- | --- | --- | --- | --- | --- | --- | --- |
|  | **n** | **GM (95% CI)** | **Overall**  **p-value** | **Pairwise**  **p-value^1^** | **n** | **GM (95% CI)** | **Overall**  **p-value** | **Pairwise**  **p-value^1^** |
| **Age (years)** | | | | | | | | |
| 32 – 39 | 82 | 0.020 (0.013, 0.032) | 0.15 |  | 82 | 0.003 (0.003, 0.004) | 0.47 |  |
| 40 – 44 | 111 | 0.021 (0.014, 0.029) |  |  | 111 | 0.004 (0.004, 0.005) |  |  |
| 45 – 49 | 71 | 0.040 (0.023, 0.071) |  |  | 71 | 0.004 (0.003, 0.005) |  |  |
| 50 and over | 25 | 0.025 (0.011, 0.057) |  |  | 25 | 0.004 (0.002, 0.006) |  |  |
| **Race and ethnicity^2^** | | | | | | | | |
| White | 258 | 0.022 (0.017, 0.028) | **0.03** |  | 258 | 0.004 (0.003, 0.004) | **0.008** |  |
| Other | 26 | 0.054 (0.021, 0.136) |  |  | 26 | 0.006 (0.004, 0.009) |  |  |
| **Country of Birth** | | | | | | | | |
| Canada | 248 | 0.022 (0.017, 0.028) | 0.10 |  | 248 | 0.004 (0.003, 0.004) | 0.64 |  |
| Other | 36 | 0.040 (0.018, 0.088) |  |  | 36 | 0.004 (0.003, 0.006) |  |  |
| **Body mass index (kg/m^2^)** | | | | | | | | |
| < 25 | 122 | 0.025 (0.017, 0.036) | 0.93 |  | 122 | 0.004 (0.003, 0.005) | 0.78 |  |
| 25 – 29 | 78 | 0.022 (0.014, 0.035) |  |  | 78 | 0.004 (0.003, 0.004) |  |  |
| ≥ 30 | 57 | 0.024 (0.014, 0.042) |  |  | 57 | 0.004 (0.003, 0.005) |  |  |
| **Smoking status** | | | | | | | | |
| Never | 192 | 0.021 (0.015, 0.027) | 0.11 |  | 192 | 0.004 (0.004, 0.005) | 0.14 |  |
| Former smoker | 75 | 0.038 (0.022, 0.065) |  |  | 75 | 0.003 (0.003, 0.004) |  |  |
| Current smoker | 22 | 0.025 (0.010, 0.061) |  |  | 22 | 0.004 (0.003, 0.007) |  |  |
| **Menopausal status** | | | | | | | | |
| Pre-menopause | 172 | 0.026 (0.019, 0.035) | 0.87 |  | 172 | 0.004 (0.003, 0.005) | 0.93 |  |
| Peri- or Post-menopausal | 54 | 0.023 (0.013, 0.041) |  |  | 54 | 0.004 (0.003, 0.005) |  |  |
| Using contraceptives | 46 | 0.022 (0.012, 0.043) |  |  | 46 | 0.004 (0.003, 0.005) |  |  |
| **Parity** | | | | | | | | |
| 1 | 32 | 0.032 (0.016, 0.065) | 0.23 |  | 32 | 0.003 (0.002, 0.004) | 0.52 |  |
| 2 | 148 | 0.022 (0.015, 0.031) |  |  | 148 | 0.004 (0.003, 0.005) |  |  |
| ≥ 3 | 93 | 0.028 (0.018, 0.043) |  |  | 93 | 0.004 (0.003, 0.005) |  |  |
| **Time since last pregnancy (years)** | | | | | | | | |
| ≤ 4 | 33 | 0.031 (0.015, 0.064) | 0.31 |  | 33 | 0.003 (0.002, 0.005) | 0.59 |  |
| > 4 – 8 | 98 | 0.019 (0.012, 0.029) |  |  | 98 | 0.004 (0.003, 0.005) |  |  |
| > 8 | 142 | 0.028 (0.020, 0.040) |  |  | 142 | 0.004 (0.003, 0.005) |  |  |
| **Number of children breastfed** | | | | | | | | |
| ≤ 1 | 41 | 0.034 (0.018, 0.064) | 0.64 |  | 41 | 0.004 (0.003, 0.005) | 0.74 |  |
| 2 | 123 | 0.027 (0.018, 0.040) |  |  | 123 | 0.004 (0.003, 0.005) |  |  |
| ≥ 3 | 86 | 0.023 (0.015, 0.036) |  |  | 86 | 0.004 (0.003, 0.005) |  |  |
| **Lifetime duration of breastfeeding (years)** | | | | | | | | |
| ≤ 2 | 121 | 0.026 (0.019, 0.041) | 0.92 |  | 121 | 0.003 (0.003, 0.004) | 0.07 |  |
| > 2 – 4 | 90 | 0.025 (0.016, 0.038) |  |  | 90 | 0.004 (0.004, 0.006) |  |  |
| > 4 | 39 | 0.028 (0.014, 0.055) |  |  | 39 | 0.004 (0.004, 0.007) |  |  |
| **Education** | | | | | | | | |
| College diploma or less | 70 | 0.023 (0.014, 0.037) | 0.71 |  | 70 | 0.004 (0.003, 0.004) | 0.29 |  |
| University degree | 219 | 0.025 (0.019, 0.033) |  |  | 219 | 0.004 (0.004, 0.005) |  |  |
| **Household Income ($CAD)** | | | | | | | | |
| ≤ 60,000 | 23 | 0.024 (0.011, 0.053) | 0.82 |  | 23 | 0.003 (0.002, 0.005) | 0.20 |  |
| 60 001 – 100 000 | 56 | 0.021 (0.012, 0.036) |  |  | 56 | 0.003 (0.003, 0.004) |  |  |
| > 100 000 | 204 | 0.025 (0.019, 0.034) |  |  | 204 | 0.004 (0.004, 0.005) |  |  |

^1^ Pairwise p-values are comparisons between categories and referent value and are corrected for multiple comparisons using the Bonferroni method. Pairwise comparisons were only calculated when the overall p-value was <0.05 and when there were multiple categories.

^2^ White: individuals who self-reported their race or ethnicity as White exclusively. Other: individuals who reported their race or ethnicity as anything other than White, including individuals who self-identified with more than one race or ethnicity

Supplemental Table 14 – Concentrations of 6:2 FTS (µg/L) and the sum of 17 PFAS according to sociodemographic characteristics

|  | **6:2 FTS** | | | | **Σ17PFAS** | | | |
| --- | --- | --- | --- | --- | --- | --- | --- | --- |
|  | **n** | **GM (95% CI)** | **Overall**  **p-value** | **Pairwise**  **p-value^1^** | **n** | **GM (95% CI)** | **Overall**  **p-value** | **Pairwise**  **p-value^1^** |
| **Age (years)** | | | | | | | | |
| 32 – 39 | 77 | 0.016 (0.010, 0.025) | 0.69 |  | 82 | 3.800 (3.368, 4.287) | 0.45 |  |
| 40 – 44 | 101 | 0.013 (0.008, 0.019) |  |  | 111 | 4.161 (3.751, 4.616) |  |  |
| 45 – 49 | 68 | 0.018 (0.011, 0.030) |  |  | 71 | 4.161 (3.655, 4.737) |  |  |
| 50 and over | 23 | 0.018 (0.007, 0.045) |  |  | 25 | 4.587 (3.686, 5.707) |  |  |
| **Race and ethnicity^2^** | | | | | | | | |
| White | 241 | 0.014 (0.011, 0.019) | 0.30 |  | 258 | 4.110 (3.838, 4.402) | 0.75 |  |
| Other | 23 | 0.023 (0.012, 0.044) |  |  | 26 | 3.960 (3.191, 4.915) |  |  |
| **Country of Birth** | | | | | | | | |
| Canada | 230 | 0.015 (0.011, 0.020) | 0.89 |  | 248 | 4.060 (3.786, 4.354) | 0.49 |  |
| Other | 34 | 0.014 (0.008, 0.026) |  |  | 36 | 4.353 (3.624, 5.229) |  |  |
| **Body mass index (kg/m^2^)** | | | | | | | | |
| < 25 | 111 | 0.014 (0.009, 0.021) | 0.63 |  | 122 | 4.314 (3.194, 4.754) | 0.19 |  |
| 25 – 29 | 76 | 0.016 (0.010, 0.025) |  |  | 78 | 3.734 (3.306, 4.216) |  |  |
| ≥ 30 | 50 | 0.011 (0.007, 0.018) |  |  | 57 | 4.077 (3.536, 4.700) |  |  |
| **Smoking status** | | | | | | | | |
| Never | 178 | 0.015 (0.011, 0.021) | 0.25 |  | 192 | 4.081 (3.771, 4.417) | 0.88 |  |
| Former smoker | 71 | 0.012 (0.008, 0.019) |  |  | 75 | 4.044 (3.563, 4.589) |  |  |
| Current smoker | 20 | 0.029 (0.011, 0.079) |  |  | 22 | 4.326 (3.425, 5.464) |  |  |
| **Menopausal status** | | | | | | | | |
| Pre-menopause | 163 | 0.013 (0.010, 0.018) | 0.25 |  | 172 | 3.900 (3.584, 4.244) | 0.12 |  |
| Peri- or Post-menopausal | 47 | 0.014 (0.007, 0.028) |  |  | 54 | 4.203 (3.614, 4.888) |  |  |
| Using contraceptives | 43 | 0.024 (0.013, 0.045) |  |  | 46 | 4.717 (4.005, 5.555) |  |  |
| **Parity** | | | | | | | | |
| 1 | 31 | 0.007 (0.004, 0.013) | 0.15 |  | 32 | 4.259 (3.536, 5.129) | **0.02^3^** | **-** |
| 2 | 136 | 0.016 (0.011, 0.023) |  |  | 148 | 4.304 (3.948, 4.693) |  | 0.99 |
| ≥ 3 | 86 | 0.016 (0.011, 0.023) |  |  | 93 | 3.565 (3.197, 3.976) |  | 0.32 |
| **Time since last pregnancy (years)** | | | | | | | | |
| ≤ 4 | 32 | 0.018 (0.009, 0.037) | 0.22 |  | 33 | 3.138 (2.619, 3.760) | **0.001** |  |
| > 4 – 8 | 91 | 0.018 (0.012, 0.027) |  |  | 98 | 3.784 (3.407, 4.203) |  | 0.22 |
| > 8 | 130 | 0.011 (0.008, 0.016) |  |  | 142 | 4.465 (4.092, 4.872) |  | **0.002** |
| **Number of children breastfed** | | | | | | | | |
| ≤ 1 | 38 | 0.006 (0.004, 0.011) | 0.11 |  | 41 | 4.503 (3.823, 5.304) | **0.002^4^** | **-** |
| 2 | 113 | 0.013 (0.008, 0.019) |  |  | 123 | 4.296 (3.908, 4.721) |  | 0.99 |
| ≥ 3 | 79 | 0.015 (0.010, 0.022) |  |  | 86 | 3.376 (3.015, 3.780) |  | **0.01** |
| **Lifetime duration of breastfeeding (years)** | | | | | | | | |
| ≤ 2 | 112 | 0.010 (0.007, 0.015) | 0.62 |  | 121 | 4.557 (4.147, 5.008) | **0.0002** | - |
| > 2 – 4 | 81 | 0.014 (0.009, 0.021) |  |  | 90 | 3.703 (3.319, 4.131) |  | **0.02** |
| > 4 | 37 | 0.013 (0.007, 0.024) |  |  | 39 | 3.111 (2.635, 3.674) |  | **0.0003** |
| **Education** | | | | | | | | |
| College diploma or less | 70 | 0.011 (0.007, 0.017) | 0.15 |  | 70 | 3.653 (3.208, 4.160) | 0.05 |  |
| University degree | 206 | 0.017 (0.012, 0.023) |  |  | 219 | 4.240 (3.939, 4.563) |  |  |
| **Household Income ($CAD)** | | | | | | | | |
| ≤ 60,000 | 21 | 0.020 (0.008, 0.051) | 0.71 |  | 23 | 4.378 (3.478, 5.510) | 0.40 |  |
| 60 001 – 100 000 | 52 | 0.013 (0.007, 0.023) |  |  | 56 | 3.750 (3.236, 4.346) |  |  |
| > 100 000 | 191 | 0.015 (0.011, 0.020) |  |  | 204 | 4.153 (3.844, 4.487) |  |  |

^1^ Pairwise p-values are comparisons between categories and referent value and are corrected for multiple comparisons using the Bonferroni method. Pairwise comparisons were only calculated when the overall p-value was <0.05 and when there were multiple categories.

^2^ White: individuals who self-reported their race or ethnicity as White exclusively. Other: individuals who reported their race or ethnicity as anything other than White, including individuals who self-identified with more than one race or ethnicity

^3^ pairwise p = 0.02 for 2 vs. ≥ 3

^4^ pairwise p = 0.004 for 2 vs. ≥ 3

Supplemental Figure 1 – Spearman correlation heat map for per- and polyfluoroalkyl substances in serum samples from adult female participants in the MIREC-ENDO study (2018 – 2021)


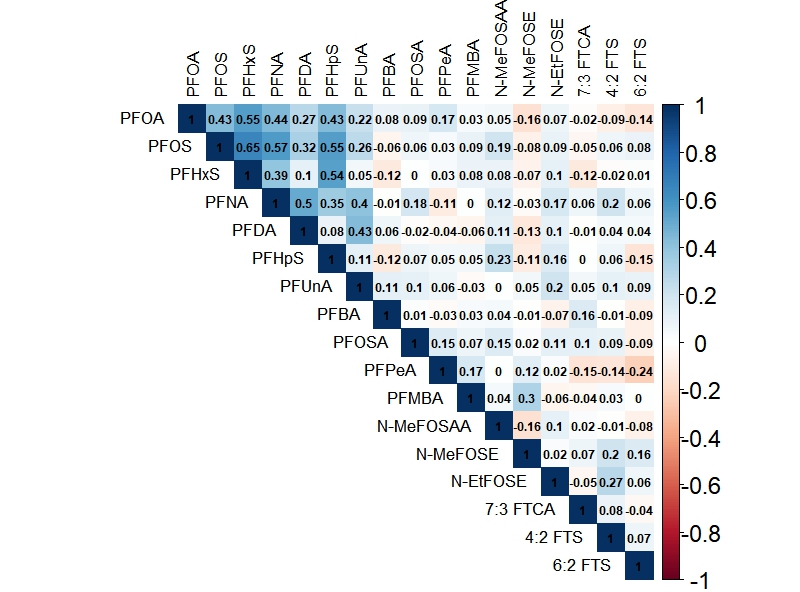

Supplement: Supplementary file 1 — Supplementary Material 1 [file 12940_2024_1085_MOESM1_ESM.docx]
